# Ecological correlates of blue whale movement behavior and its predictability in the California Current Ecosystem during the summer-fall feeding season

**DOI:** 10.1186/s40462-019-0164-6

**Published:** 2019-07-18

**Authors:** Daniel M. Palacios, Helen Bailey, Elizabeth A. Becker, Steven J. Bograd, Monica L. DeAngelis, Karin A. Forney, Elliott L. Hazen, Ladd M. Irvine, Bruce R. Mate

**Affiliations:** 1 Marine Mammal Institute and Department of Fisheries and Wildlife, Hatfield Marine Science Center, Oregon State University, Newport, OR USA; 2 Chesapeake Biological Laboratory, University of Maryland Center for Environmental Science, Solomons, MD USA; 3 Marine Mammal and Turtle Division, Southwest Fisheries Science Center, National Marine Fisheries Service, National Oceanic and Atmospheric Administration, La Jolla, CA USA; 4 Environmental Research Division, Southwest Fisheries Science Center, National Marine Fisheries Service, National Oceanic and Atmospheric Administration, Monterey, CA USA; 5 NOAA West Coast Regional Office, Long Beach, CA USA; 6 Marine Mammal and Turtle Division, Southwest Fisheries Science Center, National Marine Fisheries Service, National Oceanic and Atmospheric Administration, Moss Landing, CA USA; 7 Moss Landing Marine Laboratories, Moss Landing, CA USA; 8 Environmental Research Division, Southwest Fisheries Science Center, National Marine Fisheries Service, National Oceanic and Atmospheric Administration, Monterey, CA USA; 9 University of California Santa Cruz, Santa Cruz, CA USA; 10 Marine Mammal Institute and Department of Fisheries and Wildlife, Hatfield Marine Science Center, Oregon State University, Newport, OR USA; 11 Marine Mammal Institute and Department of Fisheries and Wildlife, Hatfield Marine Science Center, Oregon State University, Newport, OR USA; 12 Present Address: Naval Undersea Warfare Center, Newport, RI USA

**Keywords:** Satellite telemetry, State-space models, Movement behavior, Foraging behavior, Nonparametric multiplicative regression, Blue whale, *Balaenoptera musculus*, California Current Ecosystem, Decadal variability

## Abstract

**Background:**

Species distribution models have shown that blue whales (*Balaenoptera musculus*) occur seasonally in high densities in the most biologically productive regions of the California Current Ecosystem (CCE). Satellite telemetry studies have additionally shown that blue whales in the CCE regularly switch between behavioral states consistent with area-restricted searching (ARS) and transiting, indicative of foraging in and moving among prey patches, respectively. However, the relationship between the environmental correlates that serve as a proxy of prey relative to blue whale movement behavior has not been quantitatively assessed.

**Methods:**

We investigated the association between blue whale behavioral state and environmental predictors in the coastal environments of the CCE using a long-term satellite tracking data set (72 tagged whales; summer-fall months 1998–2008), and predicted the likelihood of ARS behavior at tracked locations using nonparametric multiplicative regression models. The models were built using data from years of cool, productive conditions and validated against years of warm, low-productivity conditions.

**Results:**

The best model contained four predictors: chlorophyll-*a*, sea surface temperature, and seafloor aspect and depth. This model estimated highest ARS likelihood (> 0.8) in areas with high chlorophyll-*a* levels (> 0.65 mg/m^3^), intermediate sea surface temperatures (11.6-17.5 °C), and shallow depths (< 850 m). Overall, the model correctly predicted behavioral state throughout the coastal environments of the CCE, while the validation indicated an ecosystem-wide reduction in ARS likelihood during warm years, especially in the southern portion. For comparison, a spatial coordinates model (longitude × latitude) performed slightly better than the environmental model during warm years, providing further evidence that blue whales exhibit strong foraging site fidelity, even when conditions are not conducive to successful foraging.

**Conclusions:**

We showed that blue whale behavioral state in the CCE was predictable from environmental correlates and that ARS behavior was most prevalent in regions of known high whale density, likely reflecting where large prey aggregations consistently develop in summer-fall. Our models of whale movement behavior enhanced our understanding of species distribution by further indicating where foraging was more likely, which could be of value in the identification of key regions of importance for endangered species in management considerations. The models also provided evidence that decadal-scale environmental fluctuations can drive shifts in the distribution and foraging success of this blue whale population.

**Electronic supplementary material:**

The online version of this article (10.1186/s40462-019-0164-6) contains supplementary material, which is available to authorized users.

## Background

Located off the western coast of North America, the California Current Ecosystem (CCE) boasts a rich biological productivity [1–3] and supports important populations of marine megafauna, including blue whales (*Balaenoptera musculus*) [4–7]. The Eastern North Pacific (ENP) blue whale population uses the CCE in late summer and early fall months [8–10], tracking the seasonal development of dense aggregations of euphausiid crustaceans (“krill”), their primary prey [11–13]. Krill are the key trophic link in this relatively simple food chain, consisting of phytoplankton, krill, and their predators [14, 15]. This trophic pathway was invoked by [13] to describe the sequence of events leading to the arrival of blue whales in Monterey Bay off central California in late summer, starting with springtime wind-driven coastal upwelling and followed by increased primary productivity and krill populations in spring and summer, which they termed “wind-to-whales”. This paradigm predicts that whale aggregations can be expected along this coast in areas situated downstream from upwelling centers [13], where a steep sea-floor topography, submarine canyons, and retentive circulation processes [15–19] act in concert with enhanced primary productivity and krill behavior to generate persistently high prey densities [11, 12, 20–23]. Indeed, satellite telemetry data have shown that in the CCE, blue whales aggregate in large numbers at these coastal “hotspots” [24], while ecosystem-wide studies describing blue whale distribution in relation to bathymetric and oceanographic variables have shown a clear association with the most productive habitats along the coast [9, 25–27].

Two of the most important blue whale aggregation hotspots in the CCE are found in areas of intense commercial ship traffic leading in and out of the ports of Los Angeles and San Francisco [24]. Blue whale mortality due to ship strikes has been a growing concern for the recovery of the ENP population [28–31], which is currently estimated at 1647 animals (CV = 0.07) [32], and is listed as Endangered under both the USA’s Endangered Species Act and the International Union for Conservation of Nature’s Red List [33]. Recent studies have shown that while actively engaged in feeding, whales may be less responsive to anthropogenic threats such as approaching ships [34] and military sonar operation [35], while the persistent proximity of vessels and shipping noise can result in a reduction in feeding opportunities [36, 37]. Therefore, an ecosystem-wide understanding of whale foraging behavior and its drivers would fill a critical information gap toward mitigating ship strikes and other impacts from interactions with human activities [29, 31, 38, 39].

Through the use of state-space modeling techniques, satellite telemetry data can be used to classify animal movement into simple behavioral states along with the estimation of regularly spaced locations in time along a track [40, 41]. Rapid, directed movement between locations is typically classified as “transiting mode”, while slower, localized movement is classified as “area-restricted searching (ARS) mode”. Researchers generally assume that, while in feeding areas, these ARS and transiting modes are indicative of foraging in and moving among prey patches, respectively, although these inferences have only been validated with direct measurement of behavior in a few species [42–46]. Nevertheless, behavioral state can be examined in relation to extrinsic conditions to determine whether patterns or correlations suggest an environmental basis [47–49]. Given the tight trophic coupling between blue whales and their prey during the summer-fall feeding season in the CCE, we would expect that regions of high whale density are also where ARS behavior is most prevalent.

Great strides have been made in recent years in the development and validation of habitat models (or species distribution models, SDMs) capable of predicting blue whale population abundance and distribution in the CCE from ship survey sighting data collected concurrently with bathymetric and oceanographic variables [25, 27, 50, 51]. Recently, we also applied SDMs to predict blue whale density in the CCE from satellite tracking data [26]. In this study we used this tracking data set to further investigate whether blue whale behavioral state could be similarly predicted through relationships with habitat variables as proxies for bottom-up ecological processes that favor krill aggregation. Given the relevance of behavioral context to anthropogenic threat response, an ecosystem-wide capability to predict where foraging is more likely would enhance the value of information from density models toward a more comprehensive characterization of key regions of importance for this endangered species in management considerations. Finally, an improved understanding of these species-environment relationships would also aid in better predicting the effects of climate change on the CCE ecosystem and the animal populations it supports [52, 53].

## Methods

### Blue whale tagging

The tags and tagging methodology have been documented in detail in previous papers [24, 54–56]. Briefly, ultra-high-frequency radio transmitters monitored by the Argos satellite system were attached to blue whales in three areas of the ENP (California, USA; Gulf of the California, Mexico; and in international waters of the eastern tropical Pacific) during the period 1993–2008. Tagging was conducted every year except for 1996, 1997, and 2003. Of the 182 tags deployed, 128 transmitted successfully for at least a day (d), and 104 transmitted for more than 7 d [56].

### State-space modeling of satellite telemetry data

We fitted the Bayesian switching state-space model (SSSM) developed by [40] to the unfiltered Argos locations for each whale track longer than 7 d (*n* = 104), using the software packages WinBUGS v. 1.4.3 and R v. 2.12.1 [57], as detailed in [56]. Behavioral state was specified as one of two modes, transiting (mode 1) and ARS (mode 2), based on mean turning angle and autocorrelation in speed and direction. The SSSMs ran two Markov chain Monte Carlo simulations, each for 20,000 iterations, with the first 15,000 iterations being discarded as a burn-in, and the remaining iterations being thinned, removing every fifth one to reduce autocorrelation. The result was a regularized track with one estimated location per day, along with a measure of uncertainty expressed by the posterior 95% credible limits in longitude and latitude, which accounted for the Argos location error and the movement dynamics of the animals [56]. Although only two behavioral modes were modeled, the means of the Markov chain Monte Carlo samples provided a continuous value from 1 to 2 for each location. We chose values greater than 1.75 to represent ARS mode and values lower than 1.25 to represent transiting mode, while values in between were considered “uncertain,” as has been the practice in other studies [24, 43, 56].

**Fig. 1:**
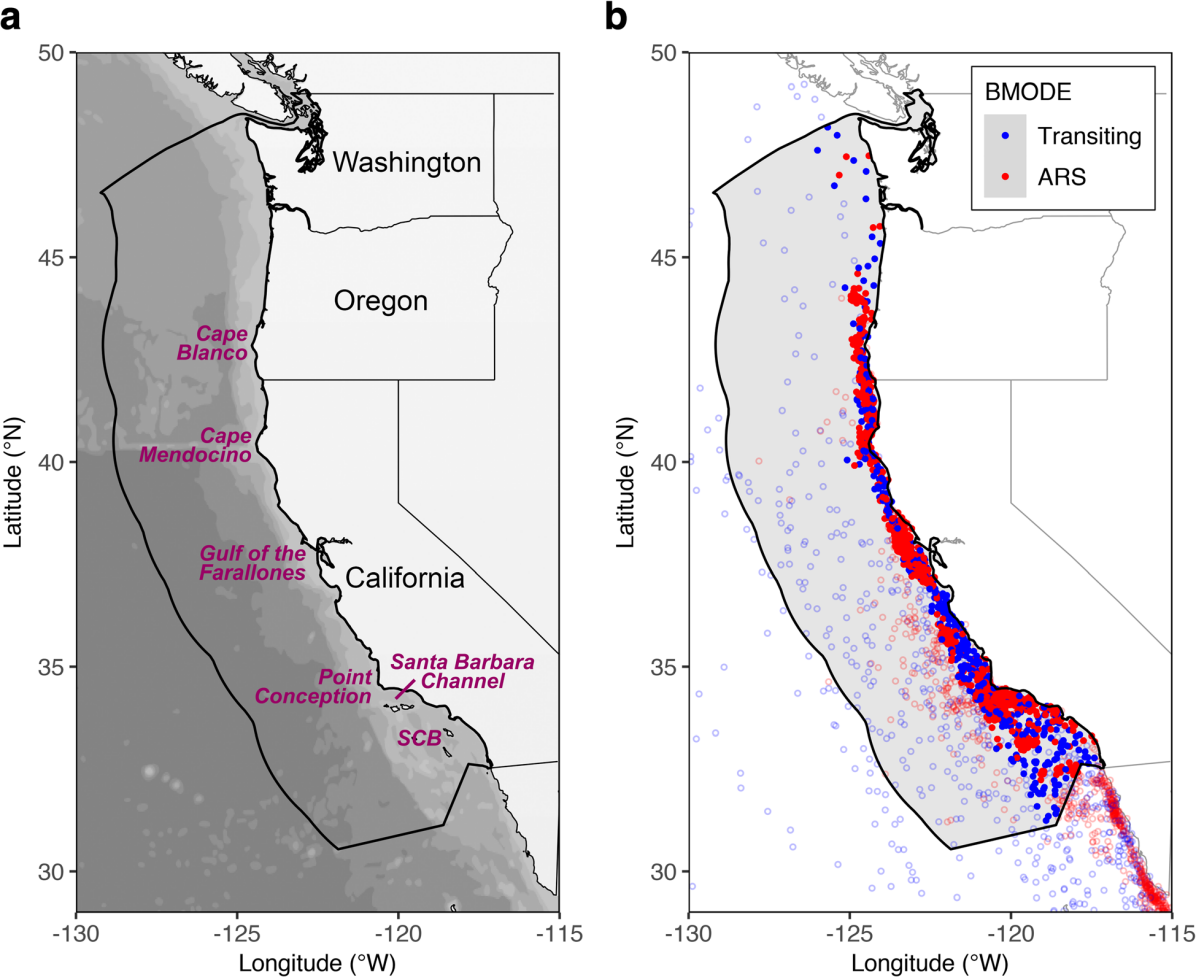
Maps of the western coast of the USA on the Pacific Ocean showing **a** the bathymetry of the study area and the names of geographic places mentioned in the text (SCB = Southern California Bight) and **b** the final set of 1808 SSSM locations in the coastal upwelling environment of the CCE (depth ≤ 2000 m) belonging to 72 Argos-monitored tags deployed over the period 1998 to 2008 (July to November months only) colored by their behavioral mode classification (BMODE, the response variable used in all NPMR models). For completeness, locations from the full tracking data set occurring within the domain of the study are shown as hollow circles. Polygon with thick black outline is the EEZ boundary

**Fig. 2:**
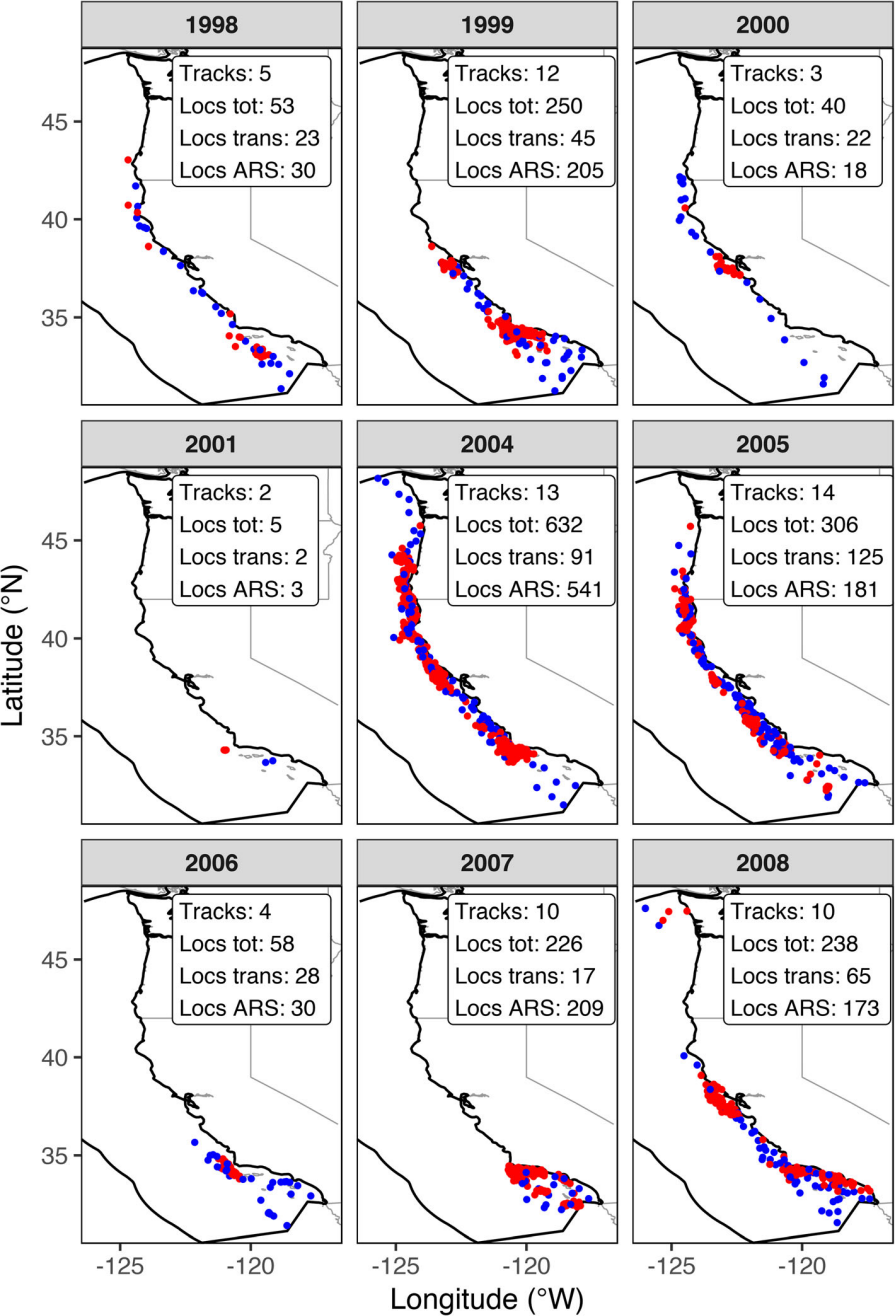
Maps of the western coast of the USA on the Pacific Ocean showing the SSSM locations used in NPMR models for each of the years of the study (1998-2008; July to November months only; depth ≤ 2,000 m), colored by their behavioral mode classification (BMODE; blue circles = transiting, red circles = ARS). For each year, the number of whale tracks and the number of locations (total, transiting, and ARS) is given in the map key. Polygon with thick black outline is the EEZ boundary

The tracking data set covered the entire migratory range of the ENP blue whale population, from the eastern tropical Pacific in the south to the Gulf of Alaska in the north [56]. However, since most of the tagging was conducted off California, the majority of the tracks were concentrated along the western coast of North America [24, 56]. Therefore, for the purpose of this study we delineated our study area using the Exclusive Economic Zone (EEZ) jurisdictional boundary of the USA on the Pacific Ocean, obtained as a polygon shapefile from the Maritime Boundaries Geodatabase [58, 59] available at the Marine Regions web site [60]. We extracted SSSM tracking data within this EEZ region (longitudinal extent: 129–117°W, latitudinal extent: 30–49°N; see Fig. 1a), with the following constraints. First, we limited the tracking data to the period after 1998, when a complete suite of environmental variables from remote sensing was available, and we further restricted the temporal scope of the study to the months from July to November to focus on the summer and fall seasons, when the whales are known to forage intensively in the coastal environments of the CCE [8, 24, 56]. To avoid bias introduced by short-lived tags toward the vicinity of the tagging locations, we only used SSSM tracks with durations longer than 14 d or a distance traveled of at least 888 km (i.e., the average transit time and separation, respectively, between ARS patches reported by [56]). We also excluded locations with SSSM estimation uncertainty exceeding 100 km in radius (based on the posterior 95% credible limits generated by the SSSM), locations with uncertain behavioral mode classification, and the last location of each track (which did not receive a behavioral classification). Lastly, to focus our study on the coastal upwelling environments where blue whales concentrate their foraging activity, we excluded portions of tracks occurring over seafloor depths greater than 2000 m (although we note that some foraging also occurs in offshore waters; see Fig. 1b). The final tracking data set contained 1808 SSSM locations belonging to 72 tagged whales, with observations in all years except 2002 and 2003 (no tag deployments took place during 2003), all occurring within a distance of 113 km from shore (Fig. 2).

### Environmental predictor data

We obtained relevant environmental variables for each SSSM location from digital elevation models and remotely sensed observations collected by oceanographic satellites available from various sources (Table 1). Variables describing the seafloor relief were depth (DEPTH), slope (SLOPE; expressed as a depth gradient), slope aspect (expressed as EASTNESS and NORTHNESS components), and distance to the 200-m isobath (or distance to the shelf break, DISTSHELF; with positive values indexing locations shoreward of the 200-m isobath and negative values indexing offshore locations). The oceanographic variables extracted included: vertical upwelling velocity (or Ekman pumping, WEKM), sea surface height (SSH), sea surface temperature (SST), and phytoplankton chlorophyll-*a* concentration (CHL). When available, these data were obtained through the web service Environmental Research Division Data Access Program (ERDDAP) [61], using the R package xtractomatic v. 3.4.1 [62], a collection of functions that permit client-side access to the data sets served by ERDDAP, to automate the process. Otherwise the data were obtained directly from the source indicated in Table 1.

**Table 1:** Details of the environmental variables obtained from digital elevation models and from oceanographic remote sensing satellites for this study, including measurement unit, abbreviation used in the text, range, spatial and temporal resolution, product name, and source. A reference to the literature is provided for product documentation or variable derivation. The original sources of the products are also given

Variable and unit	Abbrev.	Min.	Max.	Range	Spatial resolution	Temporal resolution	Product/sensor^a^	Source^b^
Bottom depth (m)	DEPTH	15.02	4669.20	4654.18	0.0083 deg	static	SRTM30_PLUS v.6.0 digital bathymetry. Reference: [65].	UCSD/SIO [66]
Bottom slope (m/km)	SLOPE	2.39	296.13	293.74	0.0083 deg	static	Derived from bathymetry using a two-dimensional Sobel gradient operator. Reference: [67].	UCSD/SIO [66]
Bottom aspect (eastness; unitless)	EASTNESS	−0.998	0.824	1.822	0.0083 deg	static	Derived from bathymetry. Reference: [68].	UCSD/SIO [66]
Bottom aspect (northness; unitless)	NORTHNESS	−0.988	0.980	1.968	0.0083 deg	static	Derived from bathymetry. Reference: [68].	UCSD/SIO [66]
Distance to shelf break (200-m isobath) (km)	DISTSHELF	− 425.23	36.01	461.24	0.0333 deg	static	ETOPO2 v.2 g 2-min Gridded Global Relief Data Reference: [69]	NOAA/NCEI [69]
Sea surface height (cm)	SSH	39.30	79.77	40.47	0.3333 deg	irregular (1–7 d)	Merged (Topex/Poseidon, ERS-1/−2, Geosat, GFO, Envisat, Jason-1/− 2). Reference: [70].	CMEMS [71]
Ekman upwelling (cm/s)	WEKM	− 1076.7	1415.0	2491.7	0.2500 deg	8 d	Seawinds/QuikSCAT, Global Ekman Current Velocity and Ekman Upwelling. Derived from wind stress. Reference: [72, 73].	ERDDAP [59], using 1-d wind-stress measurements from NASA/JPL [74] and RSS [75]
Sea surface temperature (°C)	SST	10.16	22.61	12.45	4.40 km	5 d	AVHRR Pathfinder v. 5.2 (day and night) Reference: [76].	NOAA/NCEI [77]
Chlorophyll-*a* concentration (mg/m^3^)	CHL	0.05	7.60	7.55	4.63 km	8 d	Merged (MERIS/MODIS/SeaWiFS/Polder; GSM product). Reference: [78].	GlobColour Project [79]

^a^Products not available through ERDDAP [61] were obtained directly from the source

^b^Abbreviations are defined in the text (see Abbreviations section)

In order to account for the uncertainty in location estimation by the SSSM, for each environmental variable we obtained the weighted average of the observations around each location occurring within a box defined by the SSSM posterior 95% credible limits. The observations inside this box were weighted based on distance to the SSSM location using a Bézier spatial kernel [63]. The number of observations used in this computation was dependent not only on the extent of the SSSM credible limits around each location, but also on the spatial resolution of the environmental products used, which varied from 1 km for DEPTH to 37 km for SSH (Table 2). In addition to reflecting the uncertainty in location estimation, this approach had the benefit of minimizing the number of locations with missing environmental values due to cloud cover in some of the products, had we simply obtained the single pixel value nearest to a location.

**Table 2:** Description of the NPMR models reported in this study for the building and validation sets, using locations with complete cases (no missing observations in any of the predictors). For each model, SU is the number of sample units in populated neighborhoods, *N*_*ave*_ is the realized average neighborhood size, *n*_*min*_ is the realized minimum neighborhood size (0.25 × *N*_*ave*_), log*B* is the log likelihood ratio, *B*_*ave*_ is the average contribution of a sample unit to log*B*. Additional measures of fit reported by HyperNiche include the cross-validated pseudo-*R*^*2*^ (*xR*^*2*^), the Pearson correlation (*r*) between presence/absence response data and continuous estimate of probability, and the chi-square value (χ^2^) representing the deviance comparing the model to a naive model

NPMR model	SU	*N*_*ave*_	*n*_*min*_	Log*B*	*B*_*ave*_	*xR* ^*2*^	*r*	χ^2^
Environmental								
Building	1444	93.63	23.41	26.14	1.04	0.09	0.3	100.22
Validation	364	25.10	6.28	6.39	1.04	0.09	0.3	29.42
Spatial								
Building	1444	175.80	43.95	52.20	1.03	0.06	0.3	75.14
Validation	364	46.29	11.57	10.75	1.08	0.15	0.4	49.53

Given the large latitudinal extent of our study area (~ 20 degrees), oceanographic variables susceptible to the effect of the global north-south gradient in solar heating [64] were spatially detrended by fitting a local polynomial regression (loess) smoother on latitude (degree = 1, span = 0.75) in R, such that the latitudinally detrended variables represented a “spatial anomaly” above or below the mean at a given latitude (i.e., the local deviations solely caused by dynamic oceanographic processes). The detrended variables were SSH (dtSSH) and SST (dtSST). The set of environmental variables that were initially considered as candidate predictors in the models included the original nine variables as well as the detrended versions, for a total of 11 predictors. Collinearity among the predictors was assessed with the pairwise Pearson correlation coefficient (*r*) and graphically with scatterplot matrices. Redundant predictor pairs (i.e., those exceeding the threshold |*r*| ≥ 0.7; see [80]) were considered for exclusion from multiple-predictor models based on an informal exploration of their performance in single-predictor models.

### NPMR modeling

Formal assessment of the relationship between whale behavioral mode (BMODE) and environmental predictors was conducted through nonparametric multiplicative regression (NPMR) modeling in the HyperNiche software v. 2.30 [81]. The HyperNiche implementation of NPMR is specifically designed for habitat modeling and has been shown to outperform other popular statistical techniques used in species distribution modeling like generalized additive models and random forests [82–84]. NPMR has been recently applied to animal movement data [85]. HyperNiche uses established standard practices in species distribution modeling, including leave-one-out cross-validation during fitting, overfitting controls, metrics for model selection, generation of bootstrap variability bands around fits, and randomization (Monte Carlo) tests for comparing to a null model [86–88]. A methodological overview of NPMR estimation as it applies to the context of this study is provided in the Appendix.

BMODE was encoded as a categorical binary variable indicating the absence (i.e., transiting) or presence of ARS, and the models estimated the likelihood of ARS at each SSSM location (locations with uncertain behavioral mode classification were not used in the analyses). NPMR was formulated using local mean models with Gaussian kernel weighting functions. With this configuration, the Gaussian function (which serves as a smoothing parameter) determines the weight at each location in the predictor data, while its standard deviation determines the size of the “environmental neighborhood” (n*; in units of number of locations around a target point). Model estimates are then computed at each location as the weighted average of the values of the response variable for the observations in the environmental neighborhood (with the univariate weights being combined multiplicatively), while being penalized with leave-one-out cross-validation to minimize overfitting. The corresponding formulations for these steps are shown in Eqs. 1–4 in the Appendix.

The statistics “tolerance” (the standard deviation of the Gaussian function) and “sensitivity” (the average effect size measuring the change in the level of the response for a given change in the predictors; Eq. 5 in the Appendix) indicate the relative scope and influence of the predictors, respectively. The primary metric for model selection for presence/absence response data in HyperNiche is log*B*, a log likelihood ratio expressing the improvement over a naive model (log*B* = 0). We also used *B*_*ave*_, the average contribution of a sample unit to log*B*, which is independent of sample size (see the Appendix for details).

Models were fitted using the method of free search. All models used the default overfitting controls (medium automatic settings) available in HyperNiche, and an attempt to improve the final model was made with the tuning option (see the Appendix for details). The final model was assessed with a randomization that tested the null hypothesis that the observed fit (log*B*) was no better than could be obtained by chance. Randomization was carried out by shuffling the response variable and attempting to fit the best model possible using a free search. Confidence intervals for the log*B* statistic were obtained through bootstrapping, carried out by resampling the data and fitting the model to each sample. Both the randomization and bootstrapping procedures were repeated 100 times on the data set used for model building.

### Model validation: predicting under different climatic conditions

Decadal-scale environmental variability has been hypothesized to influence long-term distributional shifts in ENP blue whales via changes in trophic linkages [89]. Given the long-term nature of our data set, we examined the possibility that blue whale movement behavior in the CCE might be in part driven by environmental fluctuations persisting across multiple years. For this purpose we used the North Pacific Gyre Oscillation (NPGO), a climate index that that closely predicts ecosystem-level changes in the ENP, with alternating phases of positive sign characterized by cool and highly productive conditions, and of negative sign characterized by warm conditions and reduced biological productivity, each lasting for several years [90]. Specifically, we built the final NPMR model on data from years dominated by a positive NPGO phase (1998–2004 and 2007–2008) and cross-validated it with data from years dominated by negative NPGO values (2005 and 2006; see Additional file 1: Figure S1) set aside for this purpose and not used for model fitting. The building data set contained 1444 SSSM locations belonging to 55 tagged whales for the positive phase of the NPGO, and the validation set contained 364 SSSM locations belonging to 18 tagged whales for the negative phase of the NPGO. This approach allowed us to test the model’s ability to predict blue whale behavioral state during different climatic regimes.

### Model assessment with binary classification

Further assessment of model performance included metrics for the success of binary classification by converting the estimated ARS likelihood into estimated presence or absence of ARS behavior under different cutoffs. To achieve the optimal binary classification, we followed the approach described by [91]. For each cutoff level of the estimates, we computed the true positive rate (TPR; i.e., the proportion of correctly identified presences, also known as sensitivity) and the true negative rate (TNR; i.e., the proportion of correctly identified absences, also known as specificity) assisted by the R package ROCR v. 1.0-7 [92], and calculated the true skill statistic (TSS) as TPR + TNR - 1 [91]. We then used the cutoff level that maximized TSS to implement the binary conversion and to compute classification statistics [prevalence, accuracy, and precision]. We also report the area under the receiver operating characteristic curve (AUC), the root-mean square error (RMSE), and the Brier score [93] as metrics of model performance.

### Accounting for spatial autocorrelation and other sources of variability

Unaccounted for spatial autocorrelation in models poses a problem for hypothesis testing because it violates the assumption of independence [94–97]. The optimized weighted averaging approach implemented by NPMR to derive model estimates (Eq. 4 in the Appendix) automatically accounts for spatial autocorrelation by estimating how much the response variable at a target location reflects response values at surrounding locations (i.e., within the environmental neighborhood), rather than treating them independently. This procedure is equivalent to the formulation implemented in generalized linear models with an autocovariate term to address spatial correlation, as reviewed by [94, 95].

Nevertheless, considering the strongly autocorrelated nature of our tracking and environmental data sets, we conducted a series of steps compatible with the NPMR framework to investigate autocorrelation. First, we fitted a purely spatial (longitude × latitude) NPMR model to capture the spatial structure in the tracking data and calculated the sample variogram [95, 98] on neighborhood sizes and predictions from both spatial and environmental models using the R package gstat v. 1.1-6 [99] to quantify the overall pattern of autocorrelation (a variogram of the Pearson residuals was of limited use given ours was a binary response). Further, we compared the estimated neighborhood sizes from the spatial and the environmental models to explore how NPMR accounted for autocorrelation in the presence of environmental predictors [94, 95].

Finally, even though NPMR modeling in HyperNiche does not have a formal way to incorporate random effects, we included individual as a categorical covariate to address potential differences in behavior driven by different animals, and offered it to the models with the rest of the environmental predictors.

## Results

### State-space modeled locations

Of the 1808 locations in the final tracking data set, 418 locations (23.1%) were classified as transiting and 1390 (76.9%) were classified as ARS mode (locations with uncertain behavioral mode classification were not used in the analyses). Blue whale locations occurred along the entire western coast of the USA (Figs. 1b and 2). ARS locations tended to cluster around three areas: Point Conception and the Santa Barbara Channel in southern California, around the Gulf of the Farallones in central California, and between Cape Mendocino and Cape Blanco in northern California and southern Oregon. Locations classified as transiting occurred between the clusters of ARS locations along the coast, as well as in the southern offshore part of the Southern California Bight (Fig. 1b).

The mean distance and speed between SSSM locations, providing an indication of the scales of blue whale daily movement within the CCE during July–November, were 39.9 km and 1.7 km/h (median = 25.7 km and 1.1 km/h, respectively). When computed by behavioral mode, the mean distance and speed for transiting locations was 81.5 km and 3.4 km/h, respectively (median = 78.4 km and 3.3 km/h, respectively), while the mean distance and speed for ARS locations was 27.3 km and 1.1 km/h (median = 19.9 km and 0.8 km/h, respectively).

### NPMR modeling results

Consideration of collinearity indicated that SSH and dtSSH were highly correlated (*r* = 0.86), as were DEPTH and DISTSHELF (*r* = − 0.78) (Additional file 2: Figure S2). In exploratory single-predictor NPMR models, SSH and DISTSHELF had inferior performance (in terms of log*B*) than dtSSH and DEPTH, respectively, so we retained dtSSH and DEPTH as a candidate predictors in multiple-predictor models. NORTHNESS was also excluded from multiple-predictor models due to a lack of predictive ability in the single-predictor models. Thus, multiple-predictor models were based on an initial set of eight predictors: DEPTH, SLOPE, EASTNESS, WEKM, dtSSH, SST, dtSST, and CHL.

A free search among this set of predictors identified the four-predictor model CHL × SST × EASTNESS × DEPTH as the best model, while meeting the parsimony requirements. After tuning, this model maximized the log likelihood ratio at log*B* = 26.14, with each location contributing an average of *B*_*ave*_ = 4% to the likelihood ratio (Table 2). This model’s average neighborhood size was *N*_*ave*_ = 93.63 and the minimum neighborhood size allowed for an estimate was *n*_*min*_ = 23.41 (Table 2). A randomization test for log*B* based on 100 runs provided evidence that this model performed significantly better than a null model (*p*-value = 0.01, log*B* mean = 0.07, log*B* variance = 0.40). Evaluation of fit for the log*B* statistic through bootstrap resampling with 100 runs indicated that it was quite stable, 90% of the time falling within the range 16.32–28.48 (the 5th and 95th percentiles, respectively). The median bootstrapped fit was log*B* = 22.20.

The predictors in this model had tolerances ranging from 0.30 mg/m^3^ for CHL to 571.97 m for DEPTH, which when scaled by the range of the predictor and expressed as a percentage, varied from 4 to 29% (Table 3). The response variable was most sensitive to changes in CHL (sensitivity = 0.71; i.e., a 20% change in CHL resulted in a 14% change in the likelihood of ARS) and least sensitive to changes in DEPTH (sensitivity = 0.07; Table 3).

**Table 3:** Characteristics of the predictors in the NPMR models reported in this study for the building (n = 1444) and validation (n = 364) sets. Note that the validation step used the tolerance of the predictors from the building step, while the sensitivity was computed for each model

	Environmental Predictors				Spatial Predictors	
	CHL	SST	DEPTH	EAST.	LONG.	LATI.
Building						
Minimum	0.05	10.56	18.57	−1.00	− 126.00	31.24
Maximum	7.60	22.61	1990.90	0.82	−117.41	48.17
Range	7.55	12.05	1972.30	1.82	8.60	16.93
Tolerance^a^	0.30	1.20	571.98	0.33	0.26	1.02
Tol. (%)^b^	4.0	10.0	29.0	18.0	3.0	6.0
Sensitivity^c^	0.71	0.24	0.07	0.12	1.21	0.32
Validation						
Minimum	0.11	10.16	17.97	−0.98	−124.89	31.41
Maximum	5.54	21.54	1997.60	0.67	−117.60	45.72
Range	5.42	11.38	1979.60	1.65	7.29	14.31
Tolerance^a^	0.30	1.20	571.98	0.33	0.26	1.02
Tol. (%)^b^	5.6	10.6	28.9	19.8	3.5	7.1
Sensitivity^c^	0.78	0.58	0.07	0.14	2.02	0.74

^a^Tolerance is the span covered by one standard deviation of the Gaussian weighting function, reported in the original scale of the predictor

^b^For comparison among predictors, tolerance is also divided by the range of the predictor and expressed as a percentage

^c^Sensitivity is the mean absolute difference resulting from nudging the predictors, expressed as a proportion of the range of the response variable

**Fig. 3:**
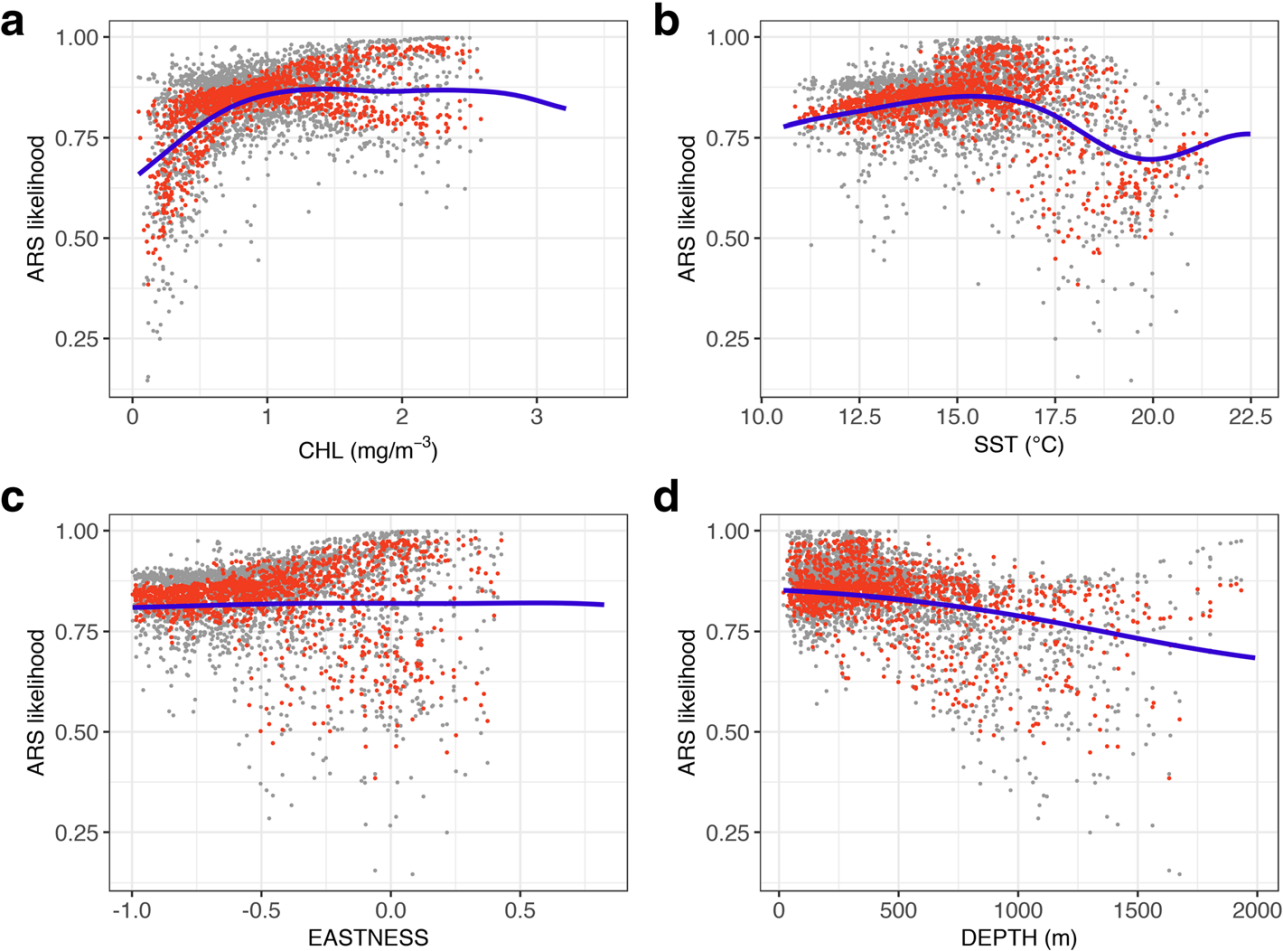
The functional responses of likelihood of ARS to **a** CHL, **b** SST, **c** EASTNESS, and **d** DEPTH in the environmental NPMR model (fitted blue curves). Also shown are the model estimates at each location (red points), and the 5th and 95th percentile variability bands obtained through 100 bootstrap samples (gray points)

The functional response of ARS likelihood with respect to CHL was described by an optimum shape, with increasing ARS likelihood at CHL levels from 0 to 1.1 mg/m^3^, followed by a broad peak from 1.1 to 2.5 mg/m^3^ and then by a slight decrease at higher CHL levels (Fig. 3a). The response to SST was also described by an optimum around 15.5 °C followed by a sharp decrease, although there was an indication of a secondary increase in ARS likelihood between 20 and 22.5 °C (Fig. 3b). The response to EASTNESS was characterized by a simple monotonic gradient, with a slight increase in ARS likelihood in the more eastward-facing slopes (although with a high variability; see Fig. 3c). Finally, the functional response of ARS likelihood to DEPTH was similarly characterized by a monotonic gradient, with highest ARS values at shallow depths (< 850 m), and decreasing values at greater depths (Fig. 3d).

**Fig. 4:**
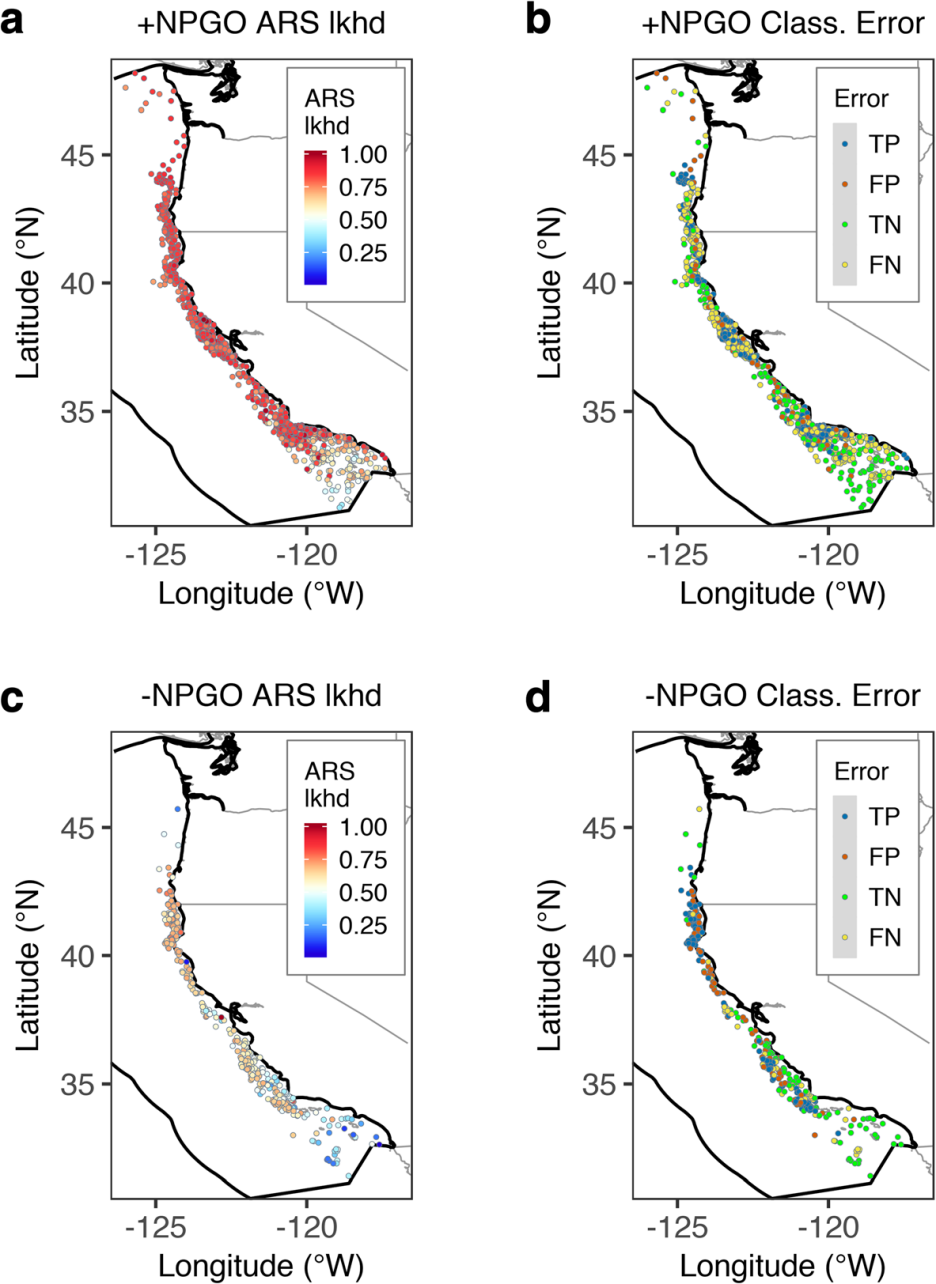
Maps of the western coast of the USA on the Pacific Ocean showing the spatial distribution of **a** 1444 SSSM locations used for model building, corresponding to tracks collected during years of positive NPGO phase (1998-2004 and 2007-2008), colored by the likelihood (lkhd) of ARS estimated by the environmental NPMR model, and **b** the corresponding classification error relative to the observations in (**a**). Panels (**c**) and (**d**) show the same results for the 364 SSSM locations in the validation set, which was collected during years of negative NPGO phase (2005 and 2006). TP = true positives, TN = true negatives, FP = false positives, FN = false negatives. Polygon with thick black outline is the EEZ boundary

The observed prevalence of ARS behavior in the building set (collected during the positive phase of the NPGO; 1998–2004 and 2007–2008; *n* = 1444) was 0.83 (Table 4). Model estimates showed widespread high ARS likelihood (above 0.8) from Point Conception in California to northern Washington (Fig. 4a). In southern California, elevated ARS likelihood was restricted to a narrow strip along the coastal margin, while the lowest ARS likelihood consistently occurred in the offshore parts of the Southern California Bight, except around offshore islands and shallow banks, where ARS likelihood was also high (Fig. 4a). In predictor space, this secondary ridge of elevated likelihood of ARS occurred in warmer temperature and shallower water (SST = 20–22.5 °C, DEPTH < 1000 m) than elsewhere in the CCE (Fig. 3b and Additional file 3: Figure S3e).

**Table 4:** Confusion matrix for the binary conversion of the likelihood of ARS estimated by the environmental NPMR model for the building (n = 1444) and validation sets (n = 364), using the cutoff value that maximized the true skill statistic (TSS_max_). FPR is the false positive rate, FNR is the false negative rate, TPR is the true positive rate, and TNR is the true negative rate. The second part of the table reports a set of performance metrics for this binary conversion, including prevalence, accuracy, precision, the area under the receiver operating characteristic curve (AUC, range: 0 to 1 with larger numbers indicating a better fit), the root-mean square error (RMSE, range: 0 to infinity with smaller numbers indicating a better fit), and the Brier score (range: 0 to 1 with lower scores indicating a better calibration of the predictions)

Confusion matrix:				
		Predictions		Classification error
		Absence	Presence	
Observations in the building set	Absence	149	77	0.34 (FPR)
	Presence	401	665	0.38 (FNR)
Observations in the validation set	Absence	17	47	0.73 (FPR)
	Presence	12	112	0.09 (FNR)
Performance metrics:				
	Building set		Validation set	
TSS_max_	0.28		0.18	
Cutoff	0.84		0.61	
Observed prevalence^a^	0.83		0.66	
Predicted prevalence^a^	0.57		0.85	
TNR (1-FPR)	0.66		0.27	
TPR (1-FNR)	0.63		0.91	
Accuracy^b^	0.63		0.69	
Precision^c^	0.90		0.70	
AUC	0.69		0.57	
RMSE	0.36		0.47	
Brier score^d^	0.13		0.22	

^a^Prevalence is estimated as: presences/total

^b^Accuracy is estimated as: (true positives + true negatives)/(obs. Presences + obs. absences)

^c^Precision is estimated as: true positives/(true positives + false positives)

^d^The Brier score is computed as the mean of the squared residuals

### Model validation: predicting under different climatic conditions

Validation of the environmental NPMR model using data from years dominated by negative NPGO values (2005 and 2006; *n* = 364; Fig. 4c and Additional file 1: Figure S1) indicated a similar degree of fit to the building set (*B*_*ave*_ = 4.5%; Table 2) despite the lower log likelihood ratio (log*B* = 6.39), which reflected the smaller sample size. This model’s average neighborhood size was *N*_*ave*_ = 25.10 and the minimum neighborhood size estimate was *n*_*min*_ = 6.28 (Table 2), both also substantially lower than for the building set. The validation set had a lower observed prevalence than the building set (0.66; Table 4).

The response variable had a similar sensitivity to changes in the predictors in the building model except for SST, which more than doubled in the validation set (sensitivity = 0.58; Table 3). An increased sensitivity to SST suggests that while the animals generally occupied the same habitat during both periods, ARS behavior was influenced by the warmer conditions during the negative phase of the NPGO. This was evident in the generalized decrease in likelihood of ARS throughout the study area, particularly in the southern half (between Point Conception and Cape Mendocino; Fig. 4c).

### Performance of binary conversion of predictions

The decrease in estimated ARS likelihood estimates for the validation set resulted in substantially different cutoffs for the optimal binary conversion into estimated presence or absence of ARS behavior between the building and validation sets (0.84 versus 0.61, respectively; Table 4 and Additional file 4: Figure S4a,). The predicted prevalence was lower than observed for the building set and higher than observed for the validation set (0.57 and 0.85, respectively; Table 4). The errors in classifying true presences and true absences were very similar (FNR = 0.38 and FPR = 0.34, respectively; Table 4) for the building set. In contrast, for the validation set the error in classifying true absences was high, while the error in classifying true presences was very low (FPR = 0.73 and FNR = 0.09, respectively; Table 4). Consequently, accuracy was slightly higher for the validation set than for the building set (0.69 and 0.63, respectively; Table 4), while precision was substantially higher for the building set than for the validation set (0.90 and 0.70, respectively; Table 4). AUC was higher for the building set than for the validation set (0.69 versus 0.57, respectively; Table 5 and Additional file 4: Figure S4b), while both RMSE and the Brier score were lower for the building set than for the validation set (RMSE 0.36 versus 0.47, respectively; Brier score = 0.13 versus 0.22, respectively; Table 4), in all cases indicating better classification performance for the building set.

**Table 5:** Confusion matrix for the binary conversion of the likelihood of ARS estimated by the spatial coordinates NPMR model for the building (*n* = 1444) and validation sets (*n* = 364), using the cutoff value that maximized the true skill statistic (TSS_max_). FPR is the false positive rate, FNR is the false negative rate, TPR is the true positive rate, and TNR is the true negative rate. The second part of the table reports a set of performance metrics for this binary conversion, including prevalence, accuracy, precision, the area under the receiver operating characteristic curve (AUC, range: 0 to 1 with larger numbers indicating a better fit), the root-mean square error (RMSE, range: 0 to infinity with smaller numbers indicating a better fit), and the Brier score (range: 0 to 1 with lower scores indicating a better calibration of the predictions)

Confusion matrix:				
		Predictions		Classification error
		Absence	Presence	
Observations in the building set	Absence	113	45	0.28 (FPR)
	Presence	404	696	0.37 (FNR)
Observations in the validation set	Absence	83	39	0.32 (FPR)
	Presence	59	146	0.29 (FNR)
Performance metrics:				
	Building set		Validation set	
TSS_max_	0.35		0.40	
Cutoff	0.90		0.65	
Observed prevalence^a^	0.87		0.63	
Predicted prevalence^a^	0.59		0.57	
TNR (1-FPR)	0.71		0.68	
TPR (1-FNR)	0.63		0.71	
Accuracy^b^	0.64		0.70	
Precision^c^	0.94		0.79	
AUC	0.71		0.72	
RMSE	0.32		0.45	
Brier score^d^	0.10		0.20	

^a^Prevalence is estimated as: presences/total

^b^Accuracy is estimated as: (true positives + true negatives)/(obs. Presences + obs. absences)

^c^Precision is estimated as: true positives/(true positives + false positives)

^d^The Brier score is computed as the mean of the squared residuals

The pattern of false negatives (i.e., ARS activity not captured by the model) in the building set was extensive throughout the study area, while the pattern of false positives (i.e., observed transiting locations that were classified as ARS by the model) was much sparser (Fig. 4b). In contrast, the proportion of false positives in the validation set was relatively high and widespread, while false negatives were very few (Fig. 4b).

### Spatial model for the autocorrelation in the tracking data

The log likelihood ratio of the tuned spatial coordinates (longitude × latitude) NPMR model fitted to the building set was twice that of the environmental model (log*B* = 52.20), while both the average neighborhood size and minimum allowed neighborhood size were 88% larger (*N*_*ave*_ = 175.80 and *n*_*min*_ = 43.95, respectively; Table 2). Each location in the spatial model contributed an average of *B*_*ave*_ = 3% to the likelihood ratio (Table 2). A randomization test for log*B* based on 100 runs provided evidence that this model performed significantly better than a null model (*p*-value = 0.01, log*B* mean = − 0.24, variance = 0.19). Evaluation of fit for the log*B* statistic through bootstrap resampling with 100 runs indicated that it was quite stable, 90% of the time falling within the range 35.32–56.19 (the 5th and 95th percentiles, respectively). The median bootstrapped fit was log*B* = 44.09 (log*B* mean = 44.92, log*B* variance = 35.08; *N*_*ave*_ mean = 265.18, *N*_*ave*_ variance = 197.17). Despite strong differences between the results of the spatial and environmental models, the binary conversion returned similar metrics of predictive performance for the two models when fitted to the building set (Tables 4 and 5, Additional file 5: Figure S5).

The tolerance value for longitude was about one fourth that of latitude (0.26 and 1.02 degrees, respectively), which when scaled by their respective ranges and expressed as a percentage, tolerances were 3% for longitude and 6% for latitude (Table 3), indicating a 1:2 scale of spatial anisotropy in longitude:latitude for the likelihood of ARS as a reflection of the more rapid decrease in ARS activity in the cross-shore direction (i.e., longitude) with increasing depth and distance from shore than in the alongshore direction. The sensitivity of the response variable to changes in latitude was correspondingly lower than it was to changes in longitude (sensitivity = 0.32 and 1.21, respectively; Table 3).

**Fig. 5:**
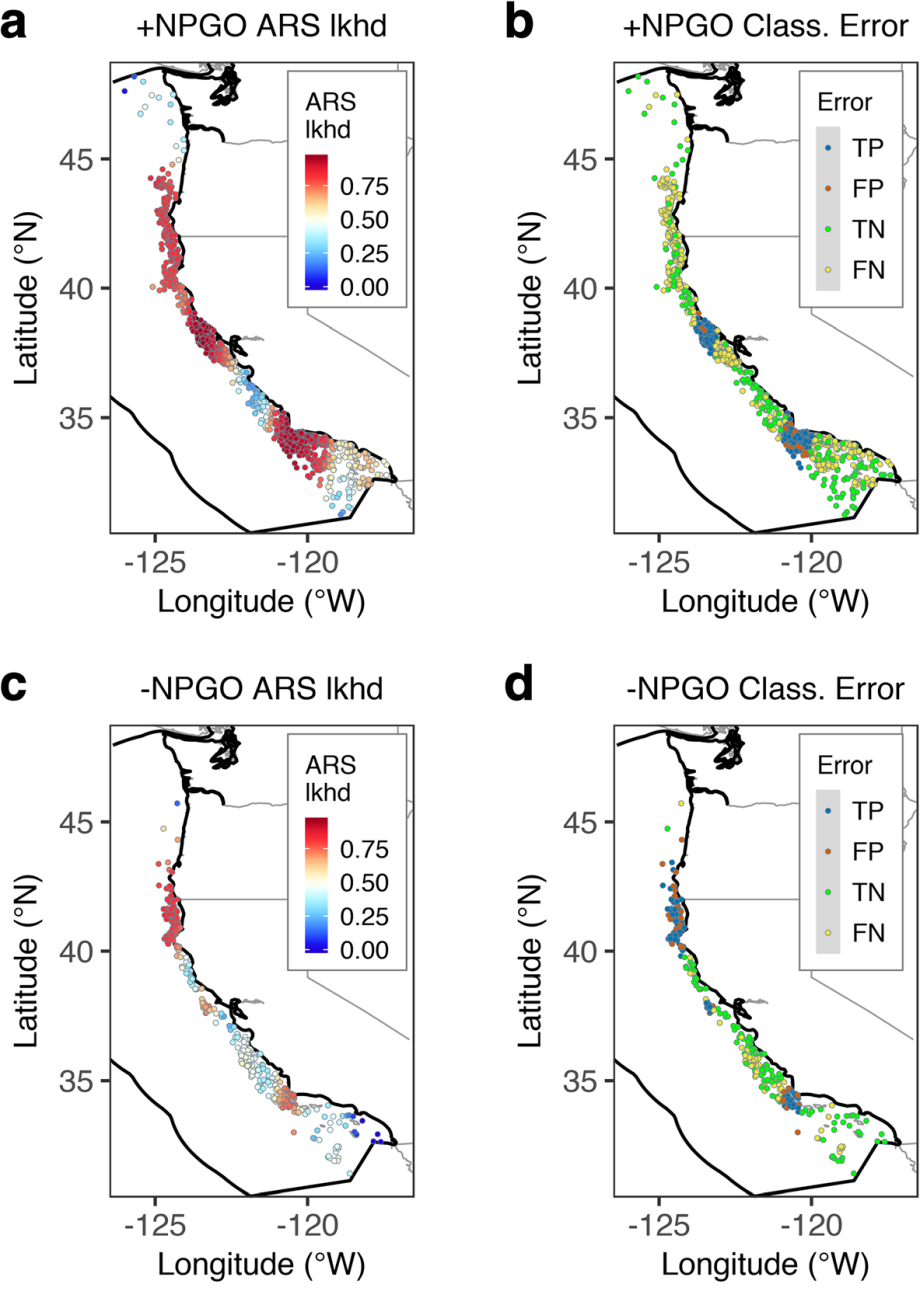
Maps of the western coast of the USA on the Pacific Ocean showing the spatial distribution of **a** 1444 SSSM locations used for model building, corresponding to tracks collected during years of positive NPGO phase (1998–2004 and 2007–2008), colored by their estimated likelihood (lkhd) of ARS by the spatial coordinates (longitude × latitude) NPMR model, and **b** the corresponding classification error relative to the observations in (**a**). Panels (**c**) and (**d**) show the same results for the 364 SSSM locations in the validation set, which was collected during years of negative NPGO phase (2005 and 2006). TP = true positives, TN = true negatives, FP = false positives, FN = false negatives. Polygon with thick black outline is the EEZ boundary

The functional responses of ARS likelihood to longitude and latitude were driven by the density of observations (Additional file 6: Figure S6), which were highly concentrated in three regions of predictor space (longitude = 125–124.5°W, 123.8–123°W, 121–119.5°W; latitude = 33–35°N, 37.2–39.5°N, 41–44°N), corresponding to an estimated high ARS likelihood in the areas around Point Conception and the Santa Barbara Channel in southern California, the Gulf of the Farallones in central California, and between Cape Mendocino and Cape Blanco in northern California and southern Oregon (Fig. 5a). The pattern of true positives and true negatives in this model’s binary classification was similarly clumped around areas of high and low likelihood of ARS, respectively (Fig. 5b, Table 5).

Strong peaks in neighborhood size in these areas of high ARS likelihood (Additional file 7: Figure S7, Additional file 8: Figure S8a and S8d) further indicated that these observations were very similar in geographic space as a result of the clumped pattern of ARS behavior in the tracking data (Fig. 1b). Therefore, the spatial coordinates NPMR model captured the pattern and scale of autocorrelation inherent in the response. In contrast, the peaks in neighborhood size and the clumping in the predictions of ARS likelihood were largely absent in the environmental NPMR model (Additional file 7: Figure S7, Additional file 8: Figure S8b and S8e), and the differences in these variables between the two models highlighted the areas where the effects of autocorrelation in the spatial model were stronger (Additional file 8: Figure S8c and S8f).

**Fig. 6:**
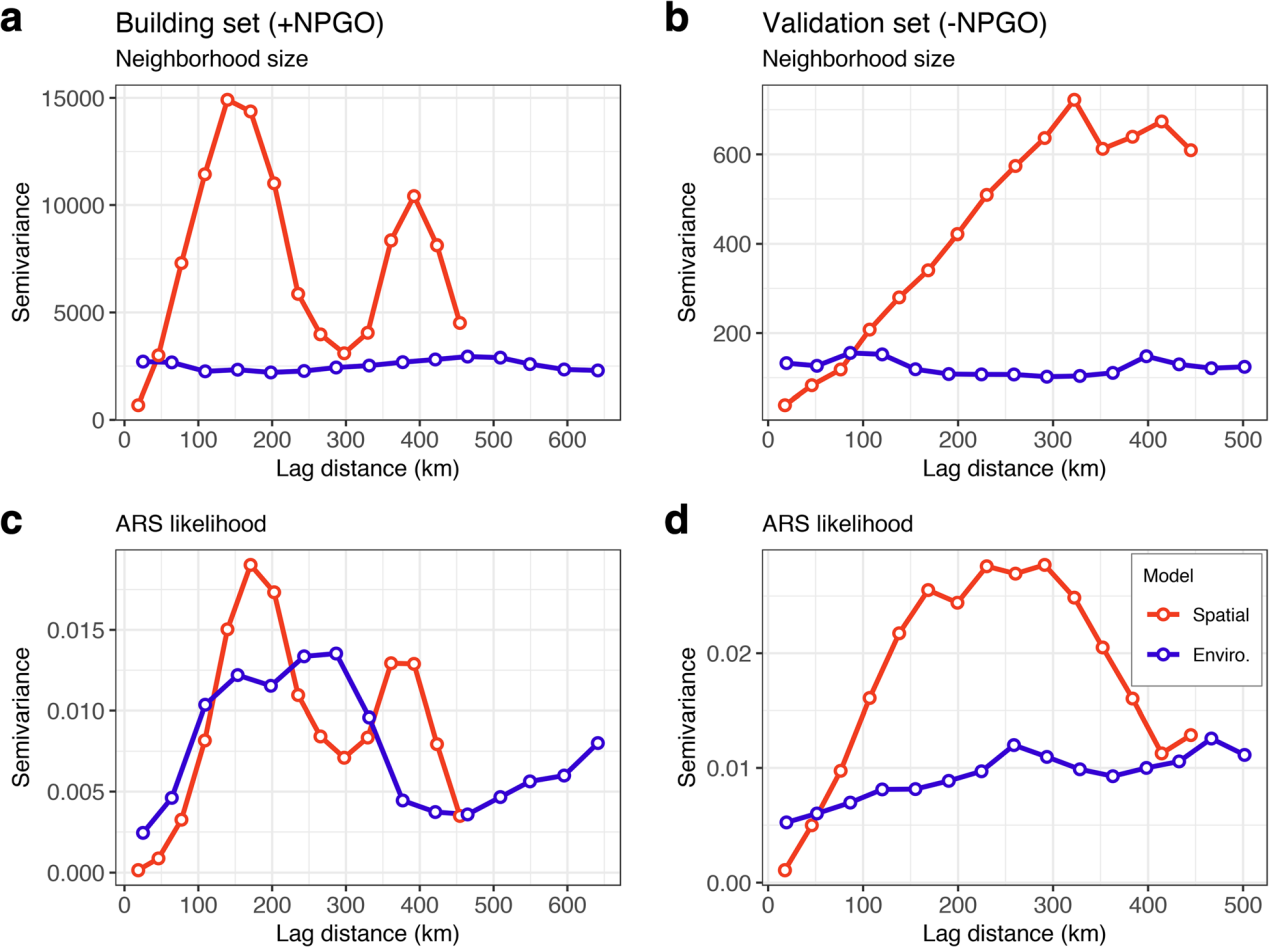
Sample variograms of neighborhood size and likelihood of ARS for NPMR models based on spatial coordinates (red line) and environmental predictors (blue line) for the building set (**a** and **c**) and the validation set (**b** and **d**)

The variogram of neighborhood size for the spatial coordinates NPMR model showed a cyclical pattern with strong autocorrelation at lags between 20 and 140 km, and secondary regions of autocorrelation between 230 and 330 km, and at 450 km, corresponding to the distances separating the three main areas of high likelihood of ARS (Fig. 6a and Additional file 8: Figure S8a and S8d). In contrast, the variogram of neighborhood size for the environmental NPMR model had much lower levels of semivariance and showed no evidence of autocorrelation at any scale (Fig. 6a). Not surprisingly, the variogram of the predicted response (ARS likelihood) for the spatial model contained the same scales of autocorrelation as neighborhood size (Fig. 6c), while the variogram of ARS likelihood for the environmental model indicated some remaining autocorrelation at scales below 100 km (attributable to measurement error or unexplained latent processes) and above 350 km (attributable to the distances separating predicted areas of high ARS likelihood by the environmental model), although the levels of semivariance were about half of those for the spatial model) (Fig. 6c).

Finally, the spatial coordinates model had a slightly better performance than the environmental model when both were fitted to the validation set (log*B* = 10.75 versus 6.39, *B*_ave_ = 8% versus 4%; Table 2). The performance metrics for the binary conversion were also slightly better for the spatial coordinates model than for the environmental model when both were fitted to the validation set (Tables 4 and 5). The variogram of neighborhood size for the spatial coordinates model showed a shift toward large-scale autocorrelation (lag distances of up to 300 km; Fig. 6b), while the levels of semivariance for the environmental model remained flat (Fig. 6b). The variograms of ARS likelihood showed that the patterns of cyclical autocorrelation observed in the building set were mostly absent from both the spatial coordinates and the environmental models when fitted to the validation set, with a slight indication of a shift toward large-scale autocorrelation (Fig. 6d).

## Discussion

Existing SDMs of blue whale population density in the CCE have been built on a variety of environmental predictors. Survey-based SDMs have included various combinations of dynamic (mixed layer depth, wind speed, sea surface salinity, SST, CHL, SSH, standard deviation of SSH) and static predictors (seafloor depth, standard deviation of seafloor depth as a proxy for slope, distance to the shelf edge) [25, 27, 50, 51]. A telemetry-based SDM of blue whale density built on the same tracking data used in this study similarly included SST, CHL, SSH standard deviation, DEPTH, and DEPTH standard deviation as predictors [26]. Although with differences in product source, temporal coverage, or resolution, all these variables variously capture aspects of surface and subsurface dynamic processes relating to upwelling and enhanced primary productivity leading to krill, while the static variables describe geomorphic features that further favor krill aggregation. Indeed, our environmental NPMR model of ARS likelihood contained a similar (but smaller) set of predictors (CHL, SST, EASTNESS, and DEPTH), perhaps because the previous models have included offshore environments (where some foraging behavior does occur; see Fig. 1b), while our model focused on the coastal environment (where blue whales forage most intensively; see Fig. 1b). Nevertheless, it is thus clear that prediction of both blue whale population density and behavioral state in the CCE requires a combination of static and dynamic variables.

The pattern of high ARS likelihood estimated by our environmental model along the coast was consistent with known regions of high whale density predicted by survey-based SDMs [25, 27, 51], as well as with the recently designated Biologically Important Areas for feeding blue whales in the CCE based on independent observations, including those associated with islands and shallow banks in offshore part of the Southern California Bight [100]. A potential region of discrepancy occurred in nearshore waters off Oregon and Washington, where our model predicted high ARS likelihood (and the telemetry-based SDM of [26] predicted high density), while survey-based SDMs predicted low whale density [25, 27, 51]. However, there were relatively few SSSM locations in this region (partly because of tag attrition, as tags were deployed in southern and central California), suggesting that this northern sector of the CCE represents favorable foraging habitat, albeit for fewer whales than in the southern sector. Recently, [19] reported important submarine canyon habitat and krill aggregations north of 45°N off the Washington coast, so additional survey and tagging efforts are needed in this region to clarify blue whale habitat use in the northern CCE, particularly given known population heterogeneity [101] and foraging site fidelity [102] among ENP blue whales.

These patterns can arise because krill distribution is not uniform along the coast, as there are multiple factors that influence its abundance, including limited supply of macro- and micronutrients required to fuel the food chain in certain areas [103–105]. In addition, strong surface currents produce additional patchiness by concentrating or dispersing zooplankton [106], while the presence of submarine canyons further determines important krill hotspots in the CCE [19]. Additionally, while in coastal waters blue whales show high prey selectivity, favoring the larger species *Thysanoessa spinifera* even when other krill species are present or dominant [12, 107].

The generalized decrease in likelihood of ARS during the negative phase of the NPGO (2005–2006), particularly south of Cape Mendocino, was the result of a decrease in neighborhood size (*N*_*ave*_ = 93.63 versus 25.10; see Table 2). This, in turn, was driven by a shift toward large-scale autocorrelation in the tracking data during this period (Fig. 6b and d), likely reflecting increased transiting and reduced ARS behavior, as evidenced by the contrast between the predictions of the spatial model for the positive and negative phases of the NPGO (see Fig. 5a and c). The slightly better performance of the spatial coordinates model over the environmental model during the negative phase of the NPGO suggests that blue whales exhibit strong foraging site fidelity, even when conditions are not conducive to successful foraging, as has been demonstrated elsewhere [27, 102]. Given that decadal-scale environmental fluctuations have been hypothesized to drive observed large-scale distributional shifts in ENP blue whales [89], our results beg the question: how long does it take for blue whales to abandon formerly reliable foraging hotspots under different climatic regimes?

Although we found that the strong autocorrelation inherent in the tracking data was implicitly addressed by the environmental NPMR model, the variograms indicated that the predictions contained some level of autocorrelation at scales below 100 km, attributable to measurement error or unexplained latent processes [98]. The daily SSSM locations indicated that while in the CCE, blue whales cover typical distances of 20 km while engaged in ARS and 78 km while in transiting, so it is possible that Argos telemetry data do not adequately resolve whale movements and behavioral states around smaller-scale features. Therefore, future studies should also conduct direct validation of blue whale behavioral states and their ecological correlates through the use of electronic tags with onboard sensors that detect individual feeding events and environmental conditions [108–110].

Despite these advances, however, habitat models will remain a valuable tool for testing and elucidating species-environment relationships within and across ecosystems [51, 111]. Given the basin-scale movements of these animals and their changing behavioral context, our models addressed an ambiguity that often arises in density SDMs: if animals are sighted in an area, are they functionally present or is the habitat irrelevant? However, since neither of these modeling approaches can simultaneously estimate both density and foraging probability, further work is needed to develop modeling approaches that integrate distributional and behavioral data, while also incorporating the various sources of uncertainty [44, 111–115].

## Conclusions

In this study, we have identified the most important large-scale environmental correlates of blue whale behavioral state in the coastal environments of the CCE. The predicted response indicated that ARS was generally consistent with foraging activities in the most biologically productive conditions and where blue whales can be expected to be most commonly found. We conclude that the environment, specifically phytoplankton chlorophyll-*a* levels, water temperature, and seafloor aspect and depth, have quantifiable effects on the movement behavior of blue whales, most likely through indirect effects on their prey. Our ecosystem-wide characterization of blue whale foraging and its drivers can be useful information in management considerations seeking to mitigate ship strikes and other anthropogenic interactions (such as entanglement in fishing gear [33]), for example through the identification of key regions of importance (and their variability in time) for this endangered species. An improved understanding of these species-environment relationships in the context of natural climatic oscillations and foraging site fidelity will also aid in better predicting the effects of climate change on the CCE ecosystem and the animal populations it supports [52, 53]. Finally, further work to integrate behavioral and distribution models for wide-ranging ocean predators such as blue whales will lead to a more complete quantification of their ecology and the risk from anthropogenic activities.

### Additional files

Additional file 1: **Figure S1.** Monthly values of the North Pacific Gyre Oscillation (NPGO) index for the period 1997-2009. Positive NPGO values (cool and highly productive conditions) were prevalent in most years of the study period (1998-2004 and 2007-2008), while two years (2005 and 2006) were characterized by negative NPGO values (warm conditions with reduced biological productivity). (PDF 117 kb)

Additional file 2: **Figure S2.** Pairwise scatterplot matrix of the 11 environmental variables used to build initial NPMR models. The data have been binned in the scatterplots in the lower triangle to avoid overplotting, with lighter color shading indicating higher density of observations. Represented along the diagonal is the univariate probability density for each variable. The upper triangle contains the Pearson correlation coefficient (r) between variable pairs. See Table 1 for the units of the variables. (PDF 1.91 mb)

Additional file 3: **Figure S3.** The estimated likelihood (lkhd) of ARS on the bivariate response surfaces represented by combinations of the predictors in the environmental NPMR model. Gray areas correspond to regions of the predictor space with non-existent combinations or where there was insufficient data for the model to produce an estimate based on the required minimum neighborhood size (*n*_min_ < 23.41). (PDF 774 kb)

Additional file 4: **Figure S4.** (a) Probability density of estimated ARS likelihood by the environmental NPMR model for the building (purple polygon) and the validation (orange polygon) sets, with the vertical lines indicating the cutoff value for binary conversion that maximized the true skill statistic for the respective sets. (b) The receiver operating characteristic curve for the binary classification of the predictions by the environmental NPMR model on the building set (purple curve) and the validation set (orange curve), compared to the 1:1 diagonal (black line) corresponding to a model that did no better than random. The AUC value is the area under the receiver operating characteristic curve for the respective curve. (PDF 243 kb)

Additional file 5: **Figure S5.** (a) Probability density of estimated ARS likelihood for NPMR models based on spatial coordinates (red polygon) and environmental predictors (purple polygon) sets, with the vertical lines indicating the cutoff value for binary conversion that maximized the true skill statistic for the respective models. (b) The receiver operating characteristic curve for the binary classification of the predictions by the spatial model (red curve) and the environmental predictors model (purple curve), compared to the 1:1 diagonal (black line) corresponding to a model that did no better than random. The AUC value is the area under the receiver operating characteristic curve for the respective curve. (PDF 248 kb)

Additional file 6: **Figure S6.** The functional responses of likelihood of ARS to (a) longitude and (b) latitude in the spatial NPMR model (fitted purple curves). Also shown are the model estimates at each location (red points), and the 5th and 95th percentile variability bands obtained through 100 bootstrap samples (gray points). The green portion of the fitted curve in (a) corresponds a region of the predictor where neighborhood size was below the minimum allowed as part of the parsimony controls (*n*_*min*_ < 43.95). (PDF 223 kb)

Additional file 7: **Figure S7.** Scatterplots of (a and b) neighborhood size and (c and d) likelihood of ARS as a function of longitude and latitude at each SSSM location for NPMR models based on spatial coordinates (red circles) and environmental predictors (purple circles). (PDF 668 kb)

Additional file 8: **Figure S8.** Maps of (a-c) neighborhood size and (d-f) likelihood of ARS at each SSSM location for NPMR models based on spatial coordinates (first column), environmental predictors (second column), and the difference between the two (third column). Polygon with thick black outline is the EEZ boundary. (PDF 524 kb)

## Data Availability

The data used in this study are available on Movebank (movebank.org, study name “Blue whales Eastern North Pacific 1993-2008 - Argos Data”) and are published in the Movebank Data Repository under a Creative Commons Zero license [116].

## Appendix

Nonparametric multiplicative habitat modeling (NPMR) in HyperNiche: NPMR models estimate the probability of occurrence – or in our case likelihood of ARS, at target point *v* as the weighted average of observations nearby (here referred to as sample units) in the multi-dimensional space defined by the quantitative values of the predictors (i.e., the environmental neighborhood) [82, 83]. The size of the environmental neighborhood (*n**) is determined by the standard deviation of the Gaussian function, and sample units near the target point (in environmental space) are weighted more strongly than distant ones in the average. Following the notation of [82, 83], the weight applied to a sample unit within the environmental neighborhood, relative to the target point *v*, is given by the Gaussian probability density function:

1 $$ {w}_{ij}^{\ast }={e}^{-\frac{1}{2}{\left[\left({x}_{ij}-{v}_j\right)/{s}_j\right]}^2} $$

where:

*w*^*^_*ij*_ = the univariate weight applied to sample unit *i* for predictor *j*,

**W** = *n* × *n* diagonal matrix with each diagonal element being a product of weights from each predictor variable. For sample unit *i*, the diagonal element is:

2 $$ {w}_{ii}=\prod \limits_{j=1}^m{w}_{ij}^{\ast}\kern0.5em $$

**X** = the matrix of environmental predictors with *i* = 1 to *n* rows (sample units) and *j* = 1 to *m* columns (predictors),

*x*_*ij*_ = the environmental predictor value at sample unit *i* for predictor *j*,

**v** = a row vector specifying the position of the target point *v* in the space defined by the environmental predictors, with *j* = 1 to *m* columns,

*v*_*j*_ = the target point for predictor *j*, and.

*s*_*j*_ = the standard deviation of the Gaussian weighting function for predictor *j*, applied such that the full range of observed values for that predictor falls over six standard deviations. (Essentially, *s*_*j*_ functions as a smoothing parameter, which in the context of NPMR is termed “bandwidth” or “tolerance”).

The size of the neighborhood, *n*^*^_*i*_, from which each estimate of the response variable *ŷ*_*v*_ is calculated, is the sum of weights for sample units:

3 $$ {n}_i^{\ast }=\sum \limits_{i=1,}^n\left(\prod \limits_{j=1}^m{w}_{ij}^{\ast}\right) $$

where 0 < *n*^*^_*i*_ ≤ *n*. If *n*^*^_*i*_ = 0, then no estimate is possible for that point.

The weights for individual environmental predictors are then combined multiplicatively (rather than additively, as is done, for example, by generalized additive models) into a single weight for a given sample unit. This weighted average gives an estimate of the probability of occurrence at target point *v*:

4 $$ {\hat{y}}_v=\frac{\sum \limits_{i=1,i\ne v}^n{y}_i\left(\prod \limits_{j=1}^m{w}_{ij}^{\ast}\right)}{\sum \limits_{i=1,i\ne v}^n\left(\prod \limits_{j=1}^m{w}_{ij}^{\ast}\right)}\kern0.75em $$

where:

*ŷ*_*v*_ = fitted value, i.e., the estimated probability of occurrence at target point *v*,

**y** = the response variable, a column vector of observed presences and absences in BMODE, from *i* = 1 to *n* rows, and.

*y*_*i*_ = the value of the response variable at sample unit *i*.

The notation *i* ≠ *v* indicates the leave-one-out cross-validation procedure implemented to help guard against overfitting the model.

Based on this weighted estimation approach, HyperNiche implements an iterative method of free search to identify the best model for a given number of predictors. Starting with the best model for a single predictor and seeking improvement at each step, the method allows both addition and deletion of predictors while simultaneously adjusting the tolerances (*s*_*j*_) of the weighting function in ±5% increments of the range of the predictors. An exhaustive search is made through all combinations of tolerances of the quantitative predictors to rank the models. In addition to using leave-one-out cross-validation for optimization, HyperNiche guards against overfitting during the search by implementing a series of controls, either with automatic settings (pre-specified levels are: conservative, medium, or aggressive, with medium being a reasonable default for most data sets) or with user-supplied manual settings. These controls include: requiring a model-wide minimum average neighborhood size (*N*^*^) that imposes parsimony on the width of the tolerances and in the number of predictors (default is 5% of the sample size), a minimum neighborhood size around a target point that protects against estimating a response in a region of the predictor space with insufficient data (default is 0.25 × *N*^*^), a maximum allowable number of sample units with missing estimates that controls the completeness of the fitted surfaces (default is 10% of the sample size), and a minimum data:predictor ratio, which for binary responses is the number of sample units in the least represented category (presences or absences) divided by the number of predictors in the model (the default minimum data:predictor ratio is 10). Lastly, HyperNiche enforces an improvement criterion on all models, expressed as a percentage improvement in model fit when a new predictor is added (the default minimum improvement criterion is a 5% increase in log*B*, the evaluation statistic). The search for additional predictors stops as soon as any one of these criteria is not met. As a final step, selected models can be tuned by further adjusting the tolerances to achieve minor improvements, using an increment of ±1% of the range of the predictors while applying the overfitting controls [82, 83].

For binary responses, overall model quality in NPMR is evaluated with log*B*, a log likelihood ratio expressing the improvement in predictive ability of a fitted model over a standard naive model (log*B* = 0) that estimates the probability of occurrence at a particular target point as the overall frequency of presences and absences in the data (in this sense, model evaluation with log*B* is analogous to evaluation using drop in deviance in logistic regression). The value of log*B* tends to increase with sample size, all else being equal, so in comparing models it is the relative amount of improvement from model to model what matters, not the absolute scale [82, 83]. Because log*B* is dependent on sample size, HyperNiche also reports *B*_*ave*_, the average contribution of a sample unit to log*B*, which allows for straightforward interpretation and comparison across data sets [82, 83].

For a given model, the sensitivity of the response variable to changes in the level of the individual predictors indicates their relative influence in driving the model (a high sensitivity to small adjustments indicates a greater importance). The most common formula used to estimate sensitivity is provided by [82, 83] as:

5 $$ Sensitivity=\frac{\left(\sum \limits_{i=1}^n\left|{\hat{y}}_{i+}-{\hat{y}}_i\right|+\sum \limits_{i=1}^n\left|{\hat{y}}_{i-}-{\hat{y}}_i\right|\right)}{2n\ \left|{y}_{max}-{y}_{min}\right|\ \Delta  } $$

where:

${\hat{y}}_{i+}$ = estimate of the response variable for case *i*, having increased the predictor by an arbitrarily small value Δ (say 0.1 of the range of the predictor),

${\hat{y}}_{i-}$ = estimate of the response variable for case *i*, having decreased the predictor by an arbitrarily small value Δ (say 0.1 of the range of the predictor), and.

Δ = a small difference applied to a predictor, expressed as a constant proportion of the range of a predictor.

Finally, a predictor’s tolerance indicates the width of the environmental window at which it operates (a predictor with a smaller tolerance has a narrower window and is a stronger driver in the model) [82, 83].

## Abbreviations

ARS: Area-restricted searching
AUC: Area under the receiver operating characteristic curve
BMODE: Behavioral mode
CCE: California Current Ecosystem
CHL: Phytoplankton chlorophyll-*a* levels
CMEMS: Copernicus Marine and Environment Monitoring Service
DEPTH: Seafloor depth
DISTSHELF: Distance to the 200-m isobath
dtSSH: Spatially detrended sea surface height
dtSST: Spatially detrended sea surface temperature
EASTNESS: Eastness component of slope aspect
EEZ: Exclusive Economic Zone
ENP: Eastern North Pacific
ERDDAP: Environmental Research Division Data Access Program
NASA/JPL: National Atmospheric and Space Administration, Jet Propulsion Laboratory
NOAA/NCEI: National Oceanic and Atmospheric Administration, National Centers for Environmental Information
NORTHNESS: Northness component of slope aspect
NPGO: North Pacific Gyre Oscillation
NPMR: Nonparametric multiplicative regression
RMSE: Root-mean square error
RSS: Remote Sensing Systems
SDM: Species distribution models
SLOPE: Seafloor slope
SSH: Sea surface height
SSSM: Bayesian switching state-space model
SST: Sea surface temperature
TNR: True negative rate
TPR: True positive rate
TSS: True skill statistic
UCSD/SIO: University of California San Diego, Scripps Institution of Oceanography
USA: United States of America
WEKM: Vertical upwelling velocity (or Ekman pumping)
