# Supplementary material for: Ecological correlates of blue whale movement behavior and its predictability in the California Current Ecosystem during the summer-fall feeding season
Source: Mov Ecol. 2019 Jul 18;7:26. doi: 10.1186/s40462-019-0164-6 (PMC6637557; doi:10.1186/s40462-019-0164-6)
Supplement: Supplementary file 1 — Figure S1. Monthly values of the North Pacific Gyre Oscillation (NPGO) index for the period 1997-2009. Positive NPGO values (cool and highly productive conditions) were prevalent in most years of the study period (1998-2004 and 2007-2008), while two years (2005 and 2006) were characterized by negative NPGO values (warm conditions with reduced biological productivity). (PDF 117 kb) [file 40462_2019_164_MOESM1_ESM.pdf]

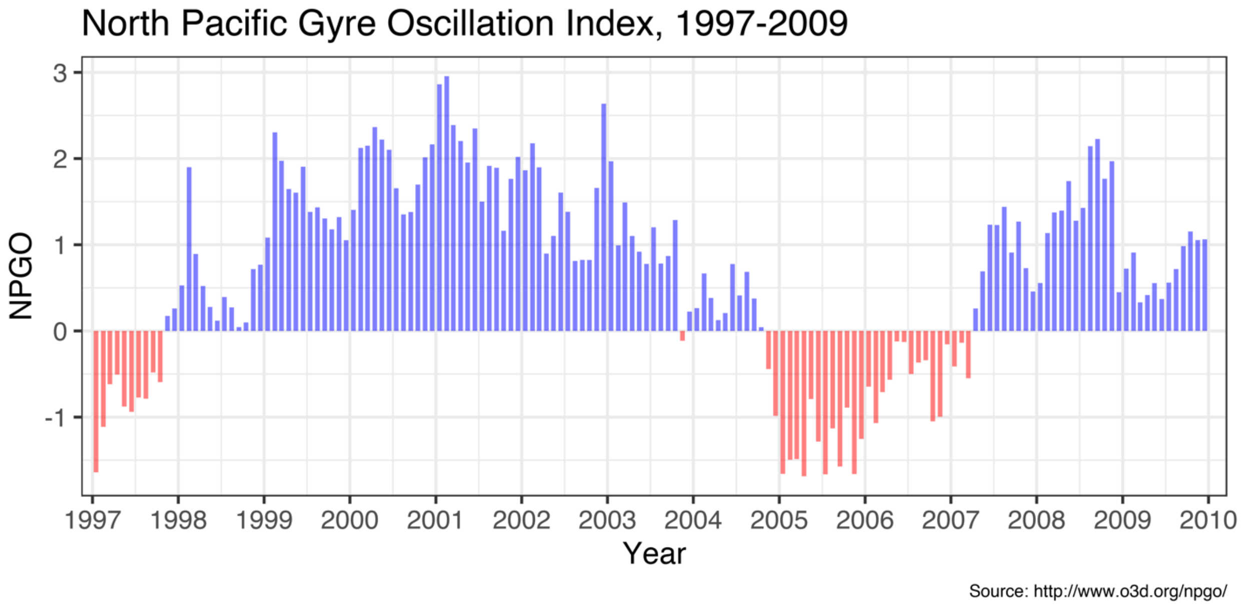

Additional file 1: Figure S1. Monthly values of the North Pacific Gyre Oscillation (NPGO) index for the period 1997-2009. Positive NPGO values (cool and highly productive conditions) were prevalent in most years of the study period (1998-2004 and 2007-2008), while two years (2005 and 2006) were characterized by negative NPGO values (warm conditions with reduced biological productivity).
