# Supplementary material for: Ecological correlates of blue whale movement behavior and its predictability in the California Current Ecosystem during the summer-fall feeding season
Source: Mov Ecol. 2019 Jul 18;7:26. doi: 10.1186/s40462-019-0164-6 (PMC6637557; doi:10.1186/s40462-019-0164-6)
Supplement: Supplementary file 2 — Figure S2. Pairwise scatterplot matrix of the 11 environmental variables used to build initial NPMR models. The data have been binned in the scatterplots in the lower triangle to avoid overplotting, with lighter color shading indicating higher density of observations. Represented along the diagonal is the univariate probability density for each variable. The upper triangle contains the Pearson correlation coefficient (r) between variable pairs. See Table 1 for the units of the variables. (PDF 1.91 mb) [file 40462_2019_164_MOESM2_ESM.pdf]

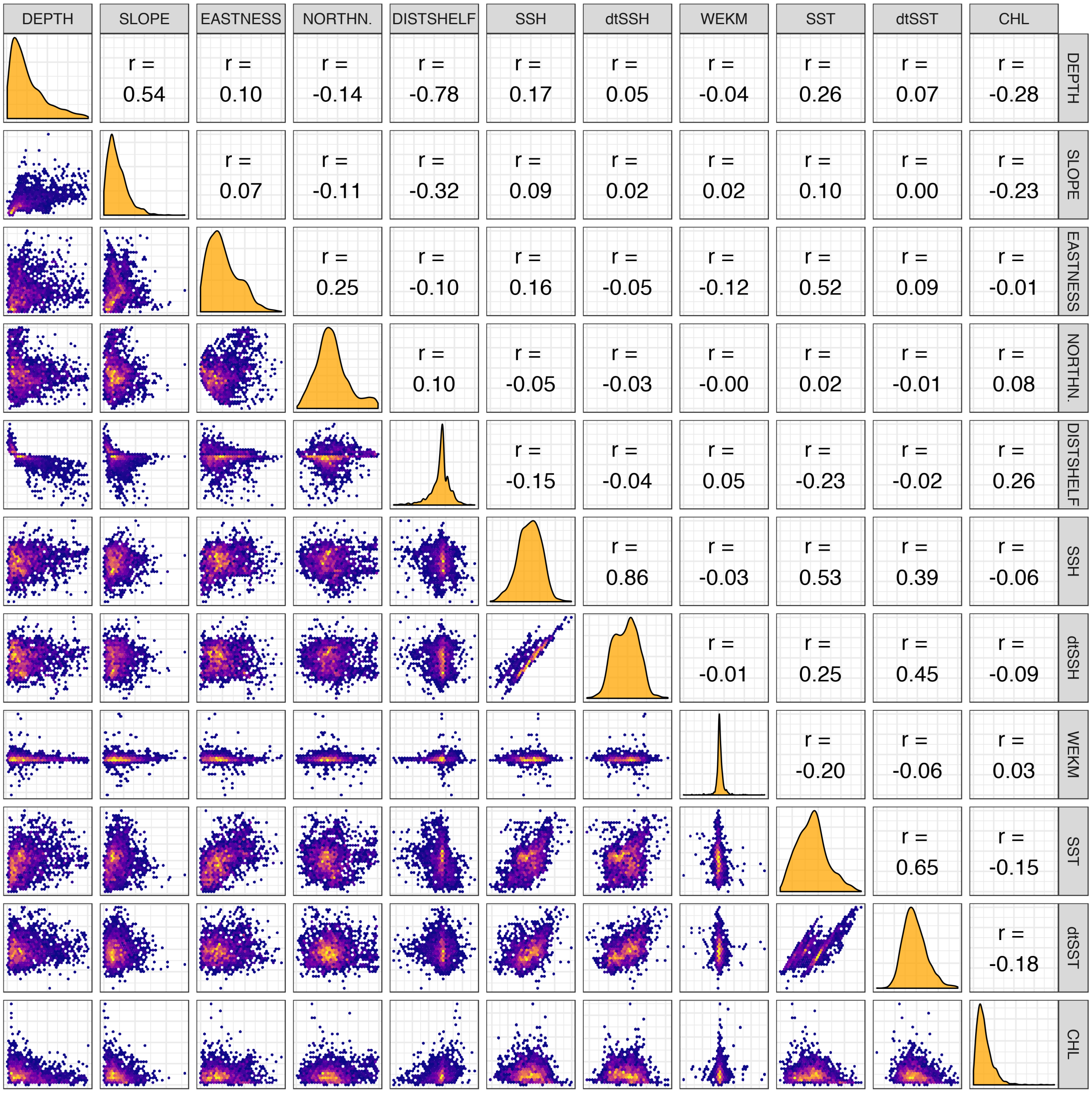

Additional file 2: Figure S2. Pairwise scatterplot matrix of the 11 environmental variables used to build initial NPMR models. The data have been binned in the scatterplots in the lower triangle to avoid overplotting, with lighter color shading indicating higher density of observations. Represented along the diagonal is the univariate probability density for each variable. The upper triangle contains the Pearson correlation coefficient (*r*) between variable pairs. See Table 1 for the units of the variables.
