# Supplementary material for: Ecological correlates of blue whale movement behavior and its predictability in the California Current Ecosystem during the summer-fall feeding season
Source: Mov Ecol. 2019 Jul 18;7:26. doi: 10.1186/s40462-019-0164-6 (PMC6637557; doi:10.1186/s40462-019-0164-6)
Supplement: Supplementary file 3 — Figure S3. The estimated likelihood (lkhd) of ARS on the bivariate response surfaces represented by combinations of the predictors in the environmental NPMR model. Gray areas correspond to regions of the predictor space with non-existent combinations or where there was insufficient data for the model to produce an estimate based on the required minimum neighborhood size (nmin < 23.41). (PDF 774 kb) [file 40462_2019_164_MOESM3_ESM.pdf]

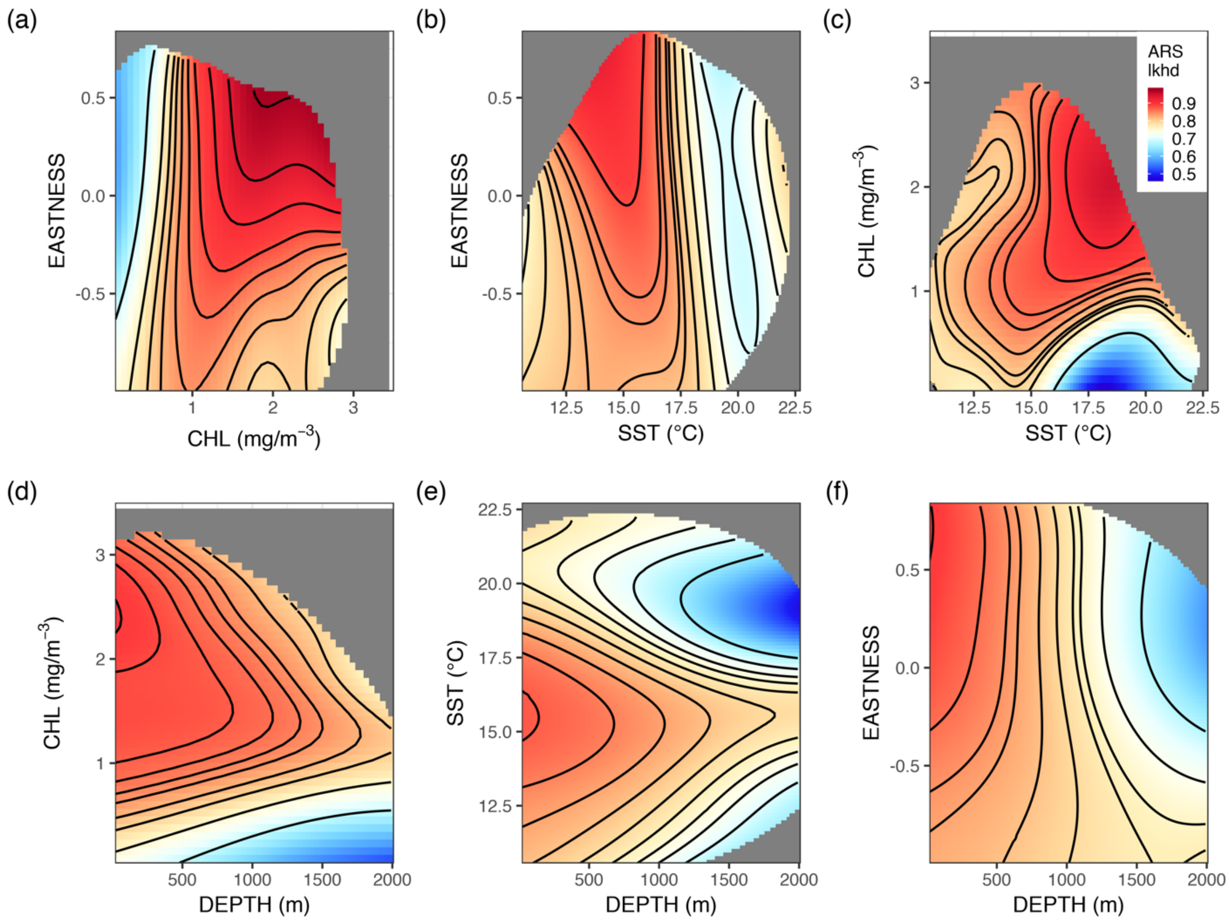

Additional file 3: Figure S3. The estimated likelihood (lkhd) of ARS on the bivariate response surfaces represented by combinations of the predictors in the environmental NPMR model. Gray areas correspond to regions of the predictor space with non-existent combinations or where there was insufficient data for the model to produce an estimate based on the required minimum neighborhood size ($n_{min} < 23.41$).
