# Supplementary material for: Ecological correlates of blue whale movement behavior and its predictability in the California Current Ecosystem during the summer-fall feeding season
Source: Mov Ecol. 2019 Jul 18;7:26. doi: 10.1186/s40462-019-0164-6 (PMC6637557; doi:10.1186/s40462-019-0164-6)
Supplement: Supplementary file 4 — Figure S4. (a) Probability density of estimated ARS likelihood by the environmental NPMR model for the building (purple polygon) and the validation (orange polygon) sets, with the vertical lines indicating the cutoff value for binary conversion that maximized the true skill statistic for the respective sets. (b) The receiver operating characteristic curve for the binary classification of the predictions by the environmental NPMR model on the building set (purple curve) and the validation set (orange curve), compared to the 1:1 diagonal (black line) corresponding to a model that did no better than random. The AUC value is the area under the receiver operating characteristic curve for the respective curve. (PDF 243 kb) [file 40462_2019_164_MOESM4_ESM.pdf]

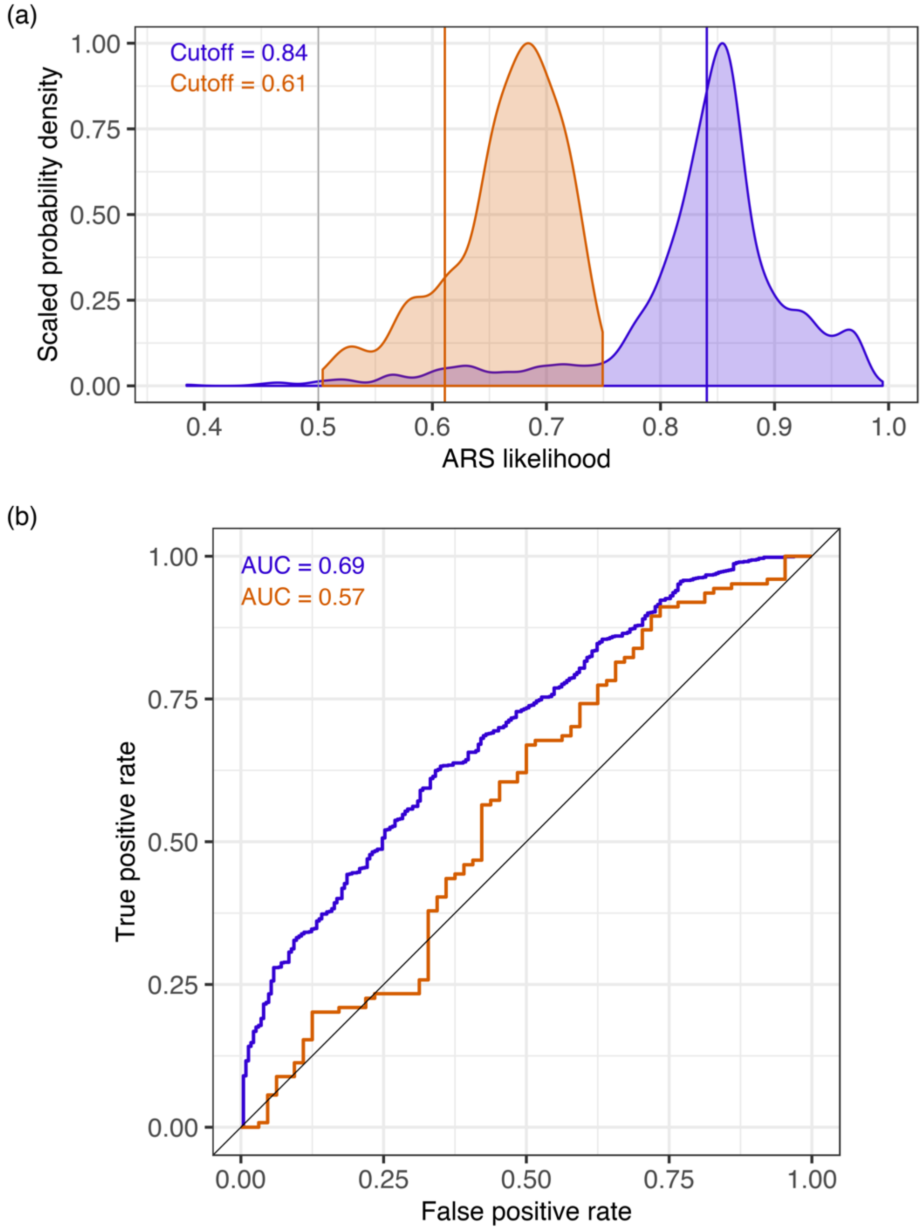

Additional file 4: Figure S4. (a) Probability density of estimated ARS likelihood by the environmental NPMR model for the building (purple polygon) and the validation (orange polygon) sets, with the vertical lines indicating the cutoff value for binary conversion that maximized the true skill statistic for the respective sets. (b) The receiver operating characteristic curve for the binary classification of the predictions by the environmental NPMR model on the building set (purple curve) and the validation set (orange curve), compared to the 1:1 diagonal (black line) corresponding to a model that did no better than random. The AUC value is the area under the receiver operating characteristic curve for the respective curve.
