# Supplementary material for: Ecological correlates of blue whale movement behavior and its predictability in the California Current Ecosystem during the summer-fall feeding season
Source: Mov Ecol. 2019 Jul 18;7:26. doi: 10.1186/s40462-019-0164-6 (PMC6637557; doi:10.1186/s40462-019-0164-6)
Supplement: Supplementary file 5 — Figure S5. (a) Probability density of estimated ARS likelihood for NPMR models based on spatial coordinates (red polygon) and environmental predictors (purple polygon) sets, with the vertical lines indicating the cutoff value for binary conversion that maximized the true skill statistic for the respective models. (b) The receiver operating characteristic curve for the binary classification of the predictions by the spatial model (red curve) and the environmental predictors model (purple curve), compared to the 1:1 diagonal (black line) corresponding to a model that did no better than random. The AUC value is the area under the receiver operating characteristic curve for the respective curve. (PDF 248 kb) [file 40462_2019_164_MOESM5_ESM.pdf]

(a)

(b)

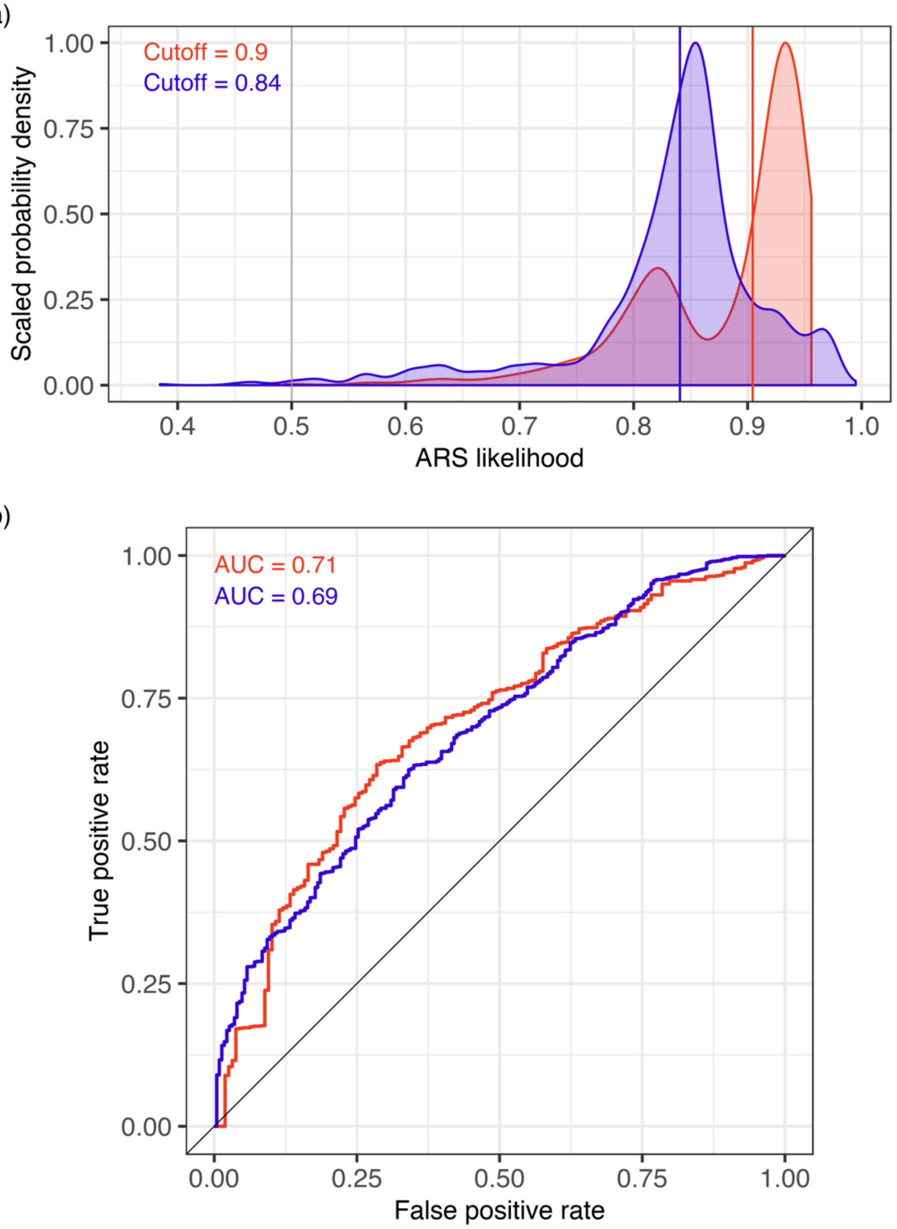

Additional file 5: Figure S5. (a) Probability density of estimated ARS likelihood for NPMR models based on spatial coordinates (red polygon) and environmental predictors (purple polygon) sets, with the vertical lines indicating the cutoff value for binary conversion that maximized the true skill statistic for the respective models. (b) The receiver operating characteristic curve for the binary classification of the predictions by the spatial model (red curve) and the environmental predictors model (purple curve), compared to the 1:1 diagonal (black line) corresponding to a model that did no better than random. The AUC value is the area under the receiver operating characteristic curve for the respective curve.
