# Supplementary material for: Ecological correlates of blue whale movement behavior and its predictability in the California Current Ecosystem during the summer-fall feeding season
Source: Mov Ecol. 2019 Jul 18;7:26. doi: 10.1186/s40462-019-0164-6 (PMC6637557; doi:10.1186/s40462-019-0164-6)
Supplement: Supplementary file 6 — Figure S6. The functional responses of likelihood of ARS to (a) longitude and (b) latitude in the spatial NPMR model (fitted purple curves). Also shown are the model estimates at each location (red points), and the 5th and 95th percentile variability bands obtained through 100 bootstrap samples (gray points). The green portion of the fitted curve in (a) corresponds a region of the predictor where neighborhood size was below the minimum allowed as part of the parsimony controls (nmin < 43.95). (PDF 223 kb) [file 40462_2019_164_MOESM6_ESM.pdf]

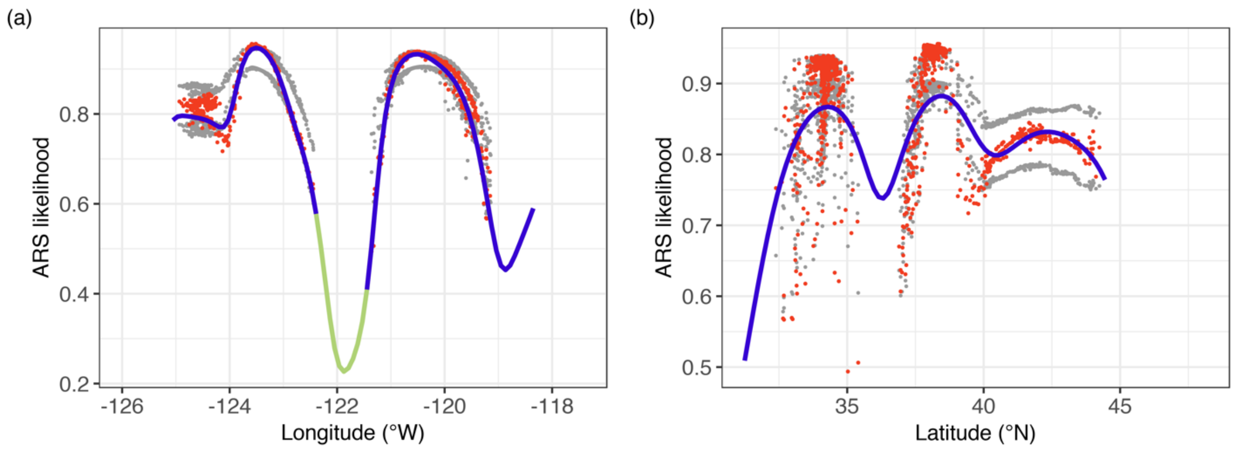

Additional file 6: Figure S6. The functional responses of likelihood of ARS to (a) longitude and (b) latitude in the spatial NPMR model (fitted purple curves). Also shown are the model estimates at each location (red points), and the 5th and 95th percentile variability bands obtained through 100 bootstrap samples (gray points). The green portion of the fitted curve in (a) corresponds a region of the predictor where neighborhood size was below the minimum allowed by the parsimony controls ($n_{min} < 43.95$).
