# Supplementary material for: Ecological correlates of blue whale movement behavior and its predictability in the California Current Ecosystem during the summer-fall feeding season
Source: Mov Ecol. 2019 Jul 18;7:26. doi: 10.1186/s40462-019-0164-6 (PMC6637557; doi:10.1186/s40462-019-0164-6)
Supplement: Supplementary file 7 — Figure S7. Scatterplots of (a and b) neighborhood size and (c and d) likelihood of ARS as a function of longitude and latitude at each SSSM location for NPMR models based on spatial coordinates (red circles) and environmental predictors (purple circles). (PDF 668 kb) [file 40462_2019_164_MOESM7_ESM.pdf]

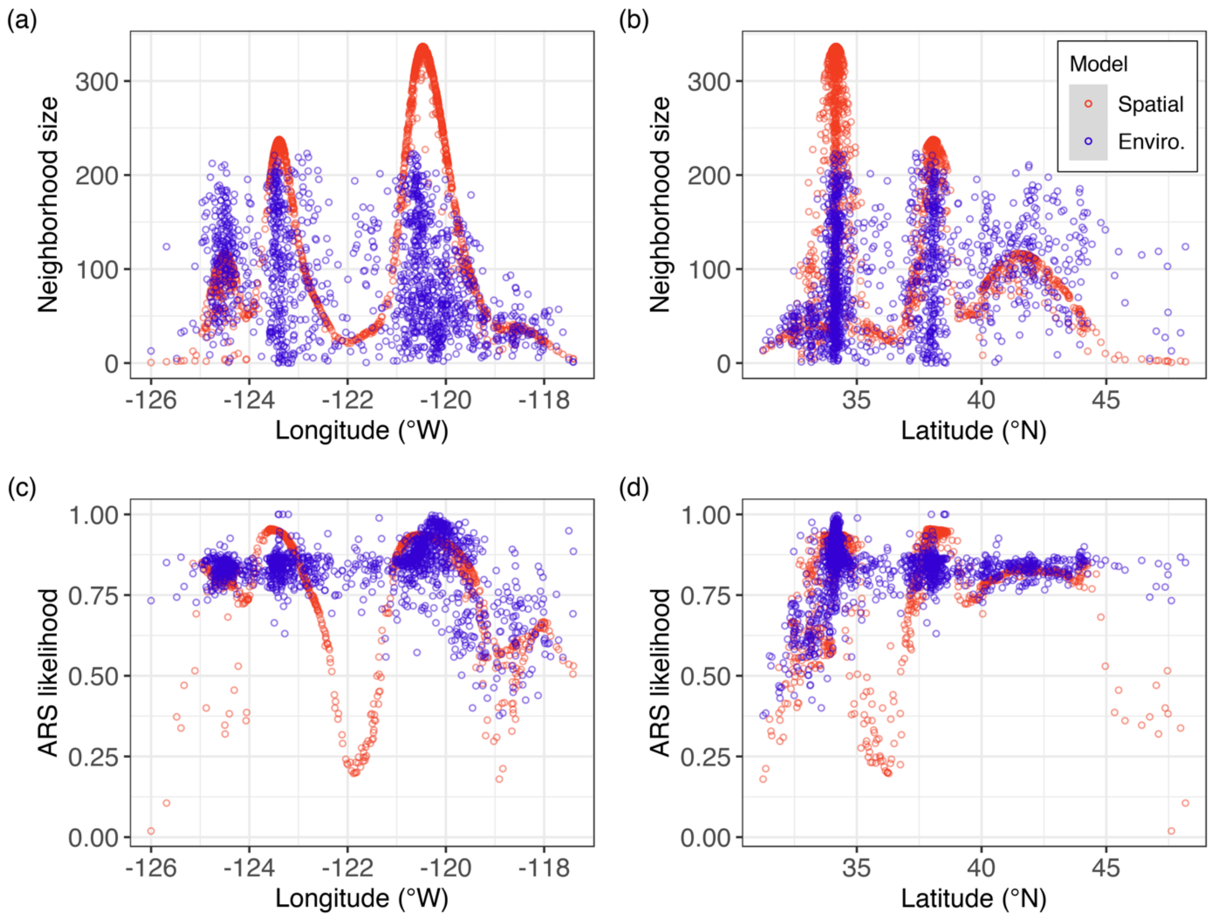

Additional file 7: Figure S7. Scatterplots of (a and b) neighborhood size and (c and d) likelihood of ARS as a function of longitude and latitude at each SSSM location for NPMR models based on spatial coordinates (red circles) and environmental predictors (purple circles).
