# Supplementary material for: Ecological correlates of blue whale movement behavior and its predictability in the California Current Ecosystem during the summer-fall feeding season
Source: Mov Ecol. 2019 Jul 18;7:26. doi: 10.1186/s40462-019-0164-6 (PMC6637557; doi:10.1186/s40462-019-0164-6)
Supplement: Supplementary file 8 — Figure S8. Maps of (a-c) neighborhood size and (d-f) likelihood of ARS at each SSSM location for NPMR models based on spatial coordinates (first column), environmental predictors (second column), and the difference between the two (third column). Polygon with thick black outline is the EEZ boundary. (PDF 524 kb) [file 40462_2019_164_MOESM8_ESM.pdf]

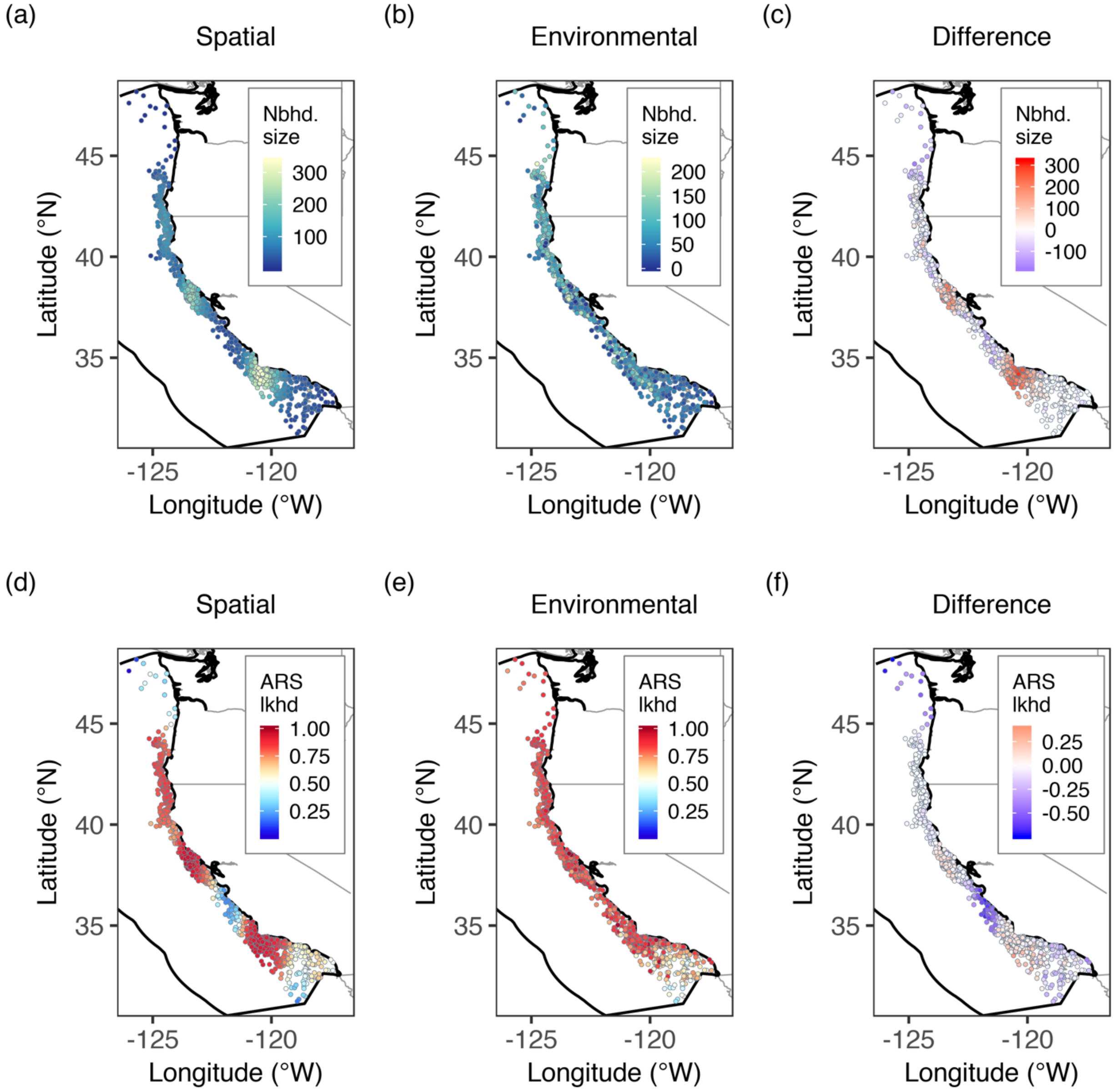

Additional file 8: Figure S8. Maps of neighborhood size (a-c) and likelihood of ARS (d-f) at each SSSM location for NPMR models based on spatial coordinates (first column), environmental predictors (second column), and the difference between the two (third column). Polygon with thick black outline is the EEZ boundary.
